# New methodologies for the detection, identification, and quantification of microplastics and their environmental degradation by-products

**DOI:** 10.1007/s11356-021-12466-z

**Published:** 2021-01-27

**Authors:** Valter Castelvetro, Andrea Corti, Greta Biale, Alessio Ceccarini, Ilaria Degano, Jacopo La Nasa, Tommaso Lomonaco, Antonella Manariti, Enrico Manco, Francesca Modugno, Virginia Vinciguerra

**Affiliations:** 1 Department of Chemistry and Industrial Chemistry, University of Pisa, 56124 Pisa, Italy; 2 CISUP - Center for the Integration of Scientific Instruments of the University of Pisa, University of Pisa, 56124 Pisa, Italy

**Keywords:** Microplastics, Polymer degradation, PET, Polyolefin, Polystyrene, Pyr-GC/MS, SIFT, VOCs

## Abstract

Sampling, separation, detection, and characterization of microplastics (MPs) dispersed in natural water bodies and ecosystems is a challenging and critical issue for a better understanding of the hazards for the environment posed by such nearly ubiquitous and still largely unknown form of pollution. There is still the need for exhaustive, reliable, accurate, reasonably fast, and cost-efficient analytical protocols allowing the quantification not only of MPs but also of nanoplastics (NPs) and of the harmful molecular pollutants that may result from degrading plastics. Here a set of newly developed analytical protocols, integrated with specialized techniques such as pyrolysis-gas chromatography-mass spectrometry (Py-GC/MS), for the accurate and selective determination of the polymers most commonly found as MPs polluting marine and freshwater sediments are presented. In addition, the results of an investigation on the low molecular weight volatile organic compounds (VOCs) released upon photo-oxidative degradation of microplastics highlight the important role of photoinduced fragmentation at a molecular level both as a potential source of hazardous chemicals and as accelerators of the overall degradation of floating or stranded plastic debris.

**Supplementary Information:**

The online version contains supplementary material available at 10.1007/s11356-021-12466-z.

## Introduction

The Mediterranean Sea is thought to contain 5–10% of the plastic debris polluting the seawaters worldwide (Suaria et al. 2016). Such pollution is potentially dangerous for aquatic life and poses serious risks for the environment as a whole, also because of the role of plastics as concentrators of molecular pollutants (Guoa and Wang 2019); as a consequence, the personal health and economic activities of the populations living along the coastal areas may be at stake in the near future (Beaumont et al. 2019). In particular, micro- and nanoplastics are being recognized as nearly ubiquitous in natural water bodies, but their actual concentration in natural waters, sediments, and biota is still largely unknown. Their identification and quantification are thus crucial for devising a global strategy for an effective and successful mitigation of this kind of pollution.

Plastic particles in the range of 1 μm to 5 mm and those in the sub-micrometer range are commonly denoted as microplastics (MPs) and nanoplastics (NPs), respectively (Cauwenberghe et al. 2015); such classification is typically based on the capture and detection thresholds of the adopted devices and techniques. MPs are found in the environment either as a result of degradation and fragmentation of larger plastic items (secondary MPs) or as particles directly produced in the form of textile fibers or of microspheres for consumer and industrial formulations (primary MPs) (Cole et al. 2011).

**Fig. 1:**
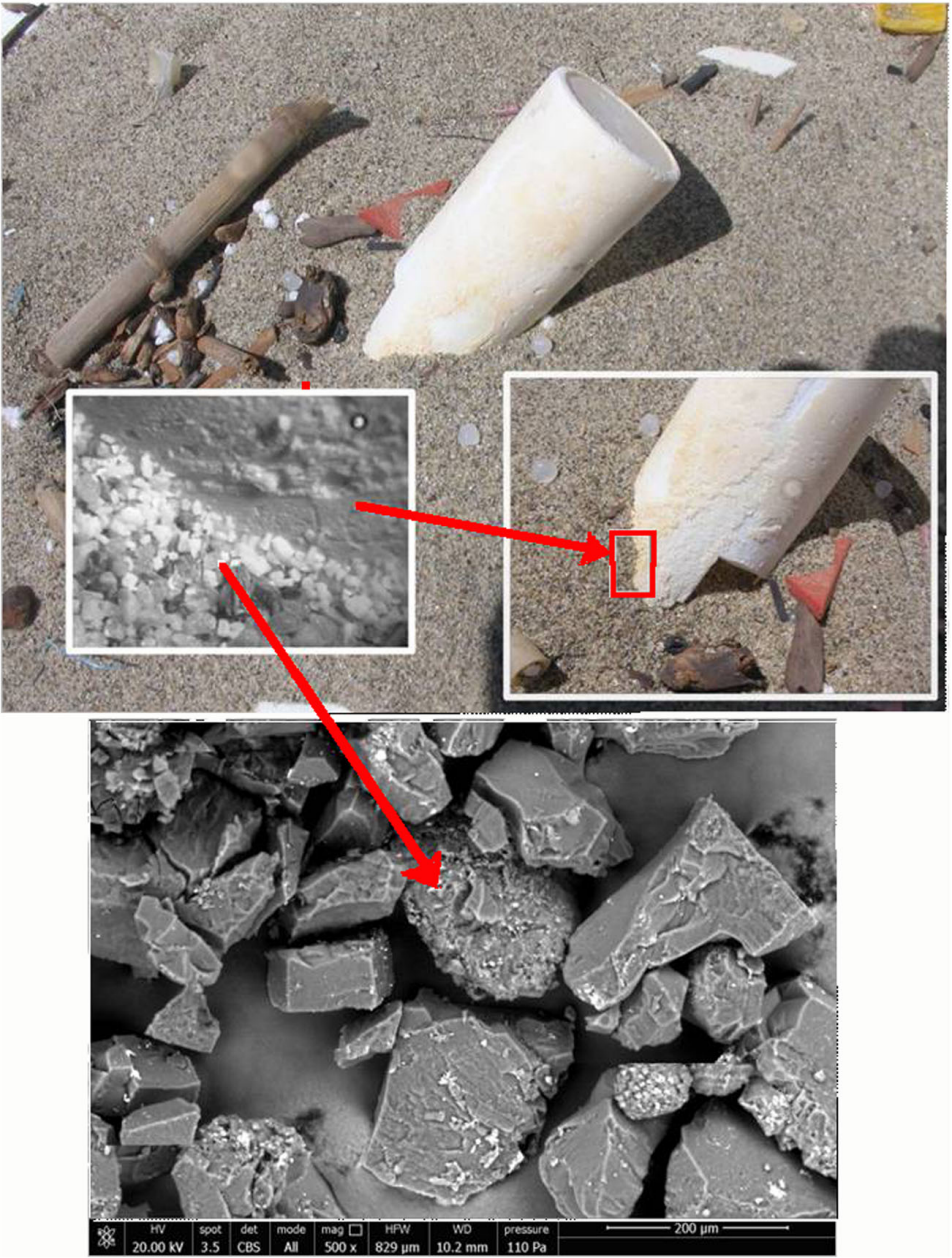
Secondary MPs resulting from surface fragmentation of a polypropylene item exposed to photo-oxidative and thermal aging (item collected by the authors from a marine beach); arrows indicate powdery plastic fragments)

The rapidly increasing number of studies about MPs has been largely focused on the isolation, detection, and mapping of their distribution in surface waters (Barnes et al. 2009) and, more recently, also in seabed, coastal, estuarine, as well as freshwater (lakes and river) sediments (Cauwenberghe et al. 2015; Hanvey et al. 2017). In fact, an estimated 80% of the total mass of marine plastic debris originates from mostly untreated wastewaters of inland urban areas (Blair et al. 2017; Cable et al. 2017). These include both larger plastic items and primary MPs such as synthetic microfibers released in laundry wastewaters. Once in the environment, all these materials undergo degradation (photo-oxidative, hydrolytic, mechanical, biological) that, depending on the type of polymeric material and exposure conditions, may result in fragmentation and size reduction down to the nanoscale (Lambert and Wagner 2016). This holds true in particular for hydrocarbon polymers such as poly(ethylene) (PE), poly(propylene) (PP), and poly(styrene (PS), largely used in packaging and single-use items and thus representing the largest fraction of the floating plastic litter (Fig. 1).

Collection of MPs from large amounts of natural waters is commonly limited to particles larger than 200–300 μm (Ivleva et al. 2017), although other devices such as pump and/or cascade filtration systems may allow to fractionally collect MPs down to 20 μm and below (Tamminga et al. 2019), particularly when high MPs concentrations make filtration of large volumes unnecessary as in the case of wastewaters (Murphy et al. 2016). When it comes to more complex environmental matrices such as sediments, sludges, or soils, sieving followed by density separation and subsequent filtration is the most common procedure for collecting microplastics as small as 1–2 μm (Missawi et al. 2020; Setälä et al. 2016). In any case, such procedures do not ensure quantitative isolation and detection of the smallest MPs, while the nano-sized particles typically go undetected. Detection errors may also result from contamination with environmental biogenic and inorganic particles. Chemical and enzymatic pre-treatments, including 30–35% hydrogen peroxide (Nuelle et al. 2014), 30% HCl, and concentrated alkaline (e.g., NaOH) solutions, have been employed to remove organic contaminants from MPs in coastal sediment samples (Imhof et al. 2012; Rocha-Santos and Duarte 2015). The chemical identification of MPs is generally based of Fourier transformed infrared (FT-IR) and Raman spectroscopies and, for smaller particles, micro-Raman and micro-FT-IR (Song et al. 2015). Pyrolysis-gas chromatography/mass spectrometry (Py-GC/MS) is also increasingly proposed as an effective and highly sensitive analytical technique allowing fast characterization with limited sample pre-treatment (Käppler et al. 2018; Matsui et al. 2020; La Nasa et al.^ 2020^); along with its modifications based, e.g., on a two-step procedure including thermal decomposition followed by adsorption onto a solid-phase device and subsequent GC/MS identification of the pyrolysis products (Duemichen et al. 2017); however, the very small amount of analyzed sample (few mg) as well as the interference from biogenic material (Harrison et al. 2011; Lobelle and Cunliffe 2011) and from persistent organic pollutants captured from the environment (Ziccardi et al. 2016) could lead to misleading interpretations of the analytical results.

In any case, both the methodologies based on separation and counting (assisted by micro-spectroscopies) and those based on bulk analysis of tiny samples or even single particles can hardly provide accurate data concerning the concentration of MPs contaminants in intrinsically variable and complex environmental samples, due to incomplete separation from the matrices and detection of the smaller particles and interference from environmental contaminants and biogenic material.

Thus, a multianalytical approach is necessary for achieving exhaustive information on the extent, distribution, and ultimately the environmental impact of MPs, including their progressive degradation eventually leading to nanometric particles and low molecular weight species.

As an alternative approach, we have been adopting wet chemical techniques such as selective solvent extraction/fractionation (for addition polymers such as polyolefins and polystyrene) or hydrolytic depolymerization (for condensation polymers such as polyethylene terephthalate, PET, and the two polyamides nylon 6 and nylon 6,6), along with quantification by liquid chromatography (size exclusion chromatography, SEC, for polymers, and reversed phase HPLC with UV and fluorescence detectors for depolymerization products), to perform an exhaustive and accurate quantitative and qualitative analysis of the total content of the main synthetic polymers present as MPs in marine and freshwater sediments (Ceccarini et al. 2018; Castelvetro et al. 2020; Castlvetro et al. 2021). This approach is being further expanded into a multianalytical platform including a range of thermally assisted GC/MS (Lomonaco et al. 2020) and SIFT-MS (selected-ion flow tube mass spectrometry, La Nasa et al. 2021) techniques aimed not only at the determination of MPs and NPs in environmental matrices but also at investigating their degradation and degradation by-products that are released as volatile organic compounds (VOCs) by the macro- and microplastic debris exposed to the environmental photo-oxidative conditions.

Here we present a survey of the results achieved so far based on the bulk analysis approach described above, which should be considered complementary to the conventional one based on particle isolation and counting as it provides more accurate quantitative results and new insights on the effects of the environmental degradation of MPs, but involves loss of information on the size, shape, color, and extent of degradation of the individual particles. Case studies concerning marine and freshwater sediments as well as laboratory studies on artificially aged MPs, focused at the present stage on polyolefins (PE, PP), polystyrene (PS), and polyethylene terephthalate (PET) as representative MPs, are also briefly presented and discussed.

## Experimental part

### Materials

Micronized virgin polypropylene (PP), low density polyethylene (LDPE), high density polyethylene (HDPE), polystyrene (PS), and polyethylene terephthalate (PET) were a kind gift from Poliplast SpA, Casnigo, Italy. All solvents and chemicals used for the extractions, hydrolytic treatment, and purification procedures described below were reagent grade commercial products used as received. HPLC-grade solvents were used for chromatographic analyses and for preparing the aqueous NaOH and HCl solutions.

### Analytical techniques

Attenuated total reflectance (ATR) FT-IR spectra were recorded as 16 scans at 4 cm^−1^ spectral resolution in the 650–4000 cm^−1^ range using a Perkin Elmer (Perkin Elmer Italia Spa, Milano, Italy) Spectrum GX spectrometer equipped with a MIRacle TM ATR accessory and a germanium crystal. A Perkin Elmer Spectrum Autoimage System microscope equipped with a germanium ATR crystal was used for micro-ATR FT-IR chemical analysis in the mid-IR region (700–4000 cm^−1^). Size exclusion chromatography (SEC) analyses were performed with an instrument consisting of a Jasco (Jasco Europe srl, Cremella, Italy) PU-2089 Plus four-channel pump with degasser, a PL gel (Polymer Laboratories) pre-column packed with polystyrene/divinylbenzene, two PL gel MIXED D columns in series, thermostated in a Jasco CO-2063 column oven, a Jasco RI 2031 Plus refractive index detector, and a Jasco UV-2077 Plus multi-channel UV spectrometer; the ChromNav Jasco software was used for data processing. Analyses were performed using trichloromethane (CHCl_3_, HPLC grade Sigma-Aldrich) as the eluent at 1 mL/min flow rate; for the analysis of PET fragments and reference PET material, the latter were previously dissolved in hexafluoroisopropanol (HFIP) and then diluted in CHCl_3_ at a 0.05 wt/vol ratio prior to the analysis. The HPLC instrument was a Jasco PU-1580 pump connected to a Jones-Genesis (Jones Chromatography Ltd., Hengoed, UK) Aq column 120 (15 cm × 4.6 mm, particle size 4 μm) and a Jasco 1575 UV-Vis detector set at 242 nm. Analyses were carried out under isocratic conditions at 0.8 mL/min flow rate of an eluent mixture consisting of HPLC-grade water (acidified with 1 wt% acetic acid, CH_3_COOH) and MeOH in a 60:40 volume ratio.

Pyrolysis-gas chromatography/mass spectrometry (Py-GC/MS) analyses were performed using an EGA/PY-3030D (Frontier Lab, Japan) multi-shot pyrolyzer coupled with a 6890N GC system with a split/splitless injection port and a mod. 5973 Agilent single quadrupole mass spectrometer (Agilent Technologies, USA); the samples (50–100 μg) placed in a stainless-steel cup were pyrolyzed at 600 °C, and the pyrolytic products were conveyed through an interface set at 280 °C into the GC injection port (also at 280 °C) operated at 1:10 split ratio. The chromatographic and mass spectrometric conditions were optimized as in Ceccarini et al. (2018). Perfluorotributylamine (PFTBA) was used for mass spectrometer tuning. MSD ChemStation (Agilent Technologies) software was used for data analysis, and the peak assignment was based on a comparison with libraries of mass spectra (NIST 8.0). For the Needle Trap Micro-Extraction coupled with GC/MS analysis (NTME-GC/MS) of VOCs, the apparatus described by Lomonaco et al. (2020) was used, comprising a 7890B GC instrument (Agilent Technologies, USA) coupled to a 7010 triple quadrupole GC/MS (Agilent Technologies, USA) equipped with a high efficiency electron ionization source operating at 70 eV. The chromatographic separation was carried out by an Agilent DB-624 ultra-inert capillary column (60 m × 0.25 mm, 1.4 μm film thickness). A Voice 200Ultra instrument (SYFT Technologies, New Zealand) was used for Select Ion Flow Tube-Mass Spectrometry (SIFT-MS) analyses performed by inserting the SIFT-MS probe directly into the headspace of the quartz vessel containing the photo-aged microplastics (flow rate 25 mL/min). Full scan analyses were performed by using H_3_O^+^ and NO^+^ reagent ions, and the products of the chemical ionization reaction were monitored by a quadrupole mass spectrometer at 60 s acquisition time, followed by data elaboration with the LabSyft 1.6.2 software. The masses used for the identification are reported in La Nasa et al. (2021).

### Microplastics photodegradation under simulated environmental aging

A RH 3000e solar box (COFOMEGRA, Milan, Italy) equipped with a Xe lamp and an outdoor filter simulating environmental conditions was used to perform artificial aging of micronized virgin polymers. Irradiation of the powders placed into 500 mL quartz tubes with polytetrafluoroethylene (PTFE) screw cap was performed at 40 °C and 750 W/m^2^ during 4 weeks; samples were collected before starting the irradiation (t0) and then with 1-week periodicity and stored in glass vials with PTFE screwcap at − 20 °C.

### Sediment sampling

**Fig. 2:**
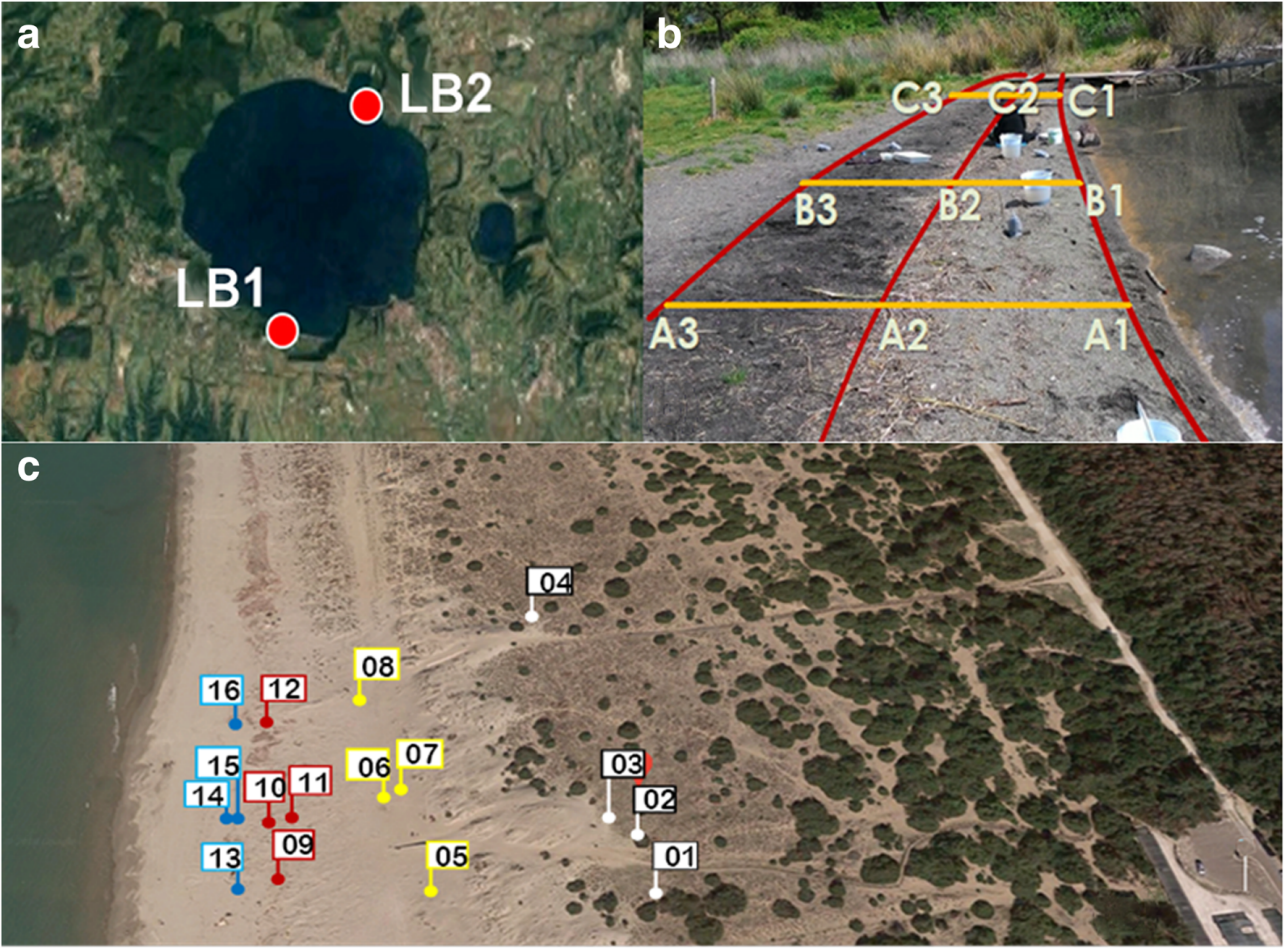
Sampling sites: (**a**) Lake Bracciano (Italy) sampling locations; (**b**) shoreline sampling spots in three transects (A–C) of the BR2 site; and (**c**) sampling spots in the marine beach of northern Tuscany (Italy), along three transects including dune (01–04, black flags), winter berm (05–08, yellow flags), summer berm (09–12, red flags), and foreshore (13–16, blue flags). Zone

Sediment samples from Lake Bracciano, Italy (LB, 42° 07′ 16″ N, 12° 13′ 55″ E), were collected near the shoreline in two sites on the opposite sides of the lake characterized by high (LB1) and low (LB2) accumulation of floating debris due to the different exposure to prevailing winds (Fig. 2a), as detailed by Corti et al. (2020). On each sampling site, nine sediment samples were collected at the intersection of three transects (A, B, and C, Fig. 2b) with the edges of three visibly recognizable storage areas parallel to the shoreline (horizons 1, 2, and 3); a metal shovel was used to collect the top 5 cm sediment within a 50 × 50 cm^2^ square area in each point.

Marine sediment samples were collected from a sandy beach in northern Tuscany, Italy (43° 80′ N, 10° 26′ E) along four transects running from the intertidal to the dune zone (Fig. 2c). Sixteen samples were collected by 25 cm core drilling using cylindrical (11 cm diameter) glass vessels in the dunes (sector A, samples 1–4), winter berm (sector B, samples 2–8), summer berm (sector C, samples 9–12), and foreshore (sector D, samples 13–16) zone in each transect, as detailed by Ceccarini et al. (2018).

### Isolation and characterization of macro- and microplastics in sediment samples

**Fig. 3:**
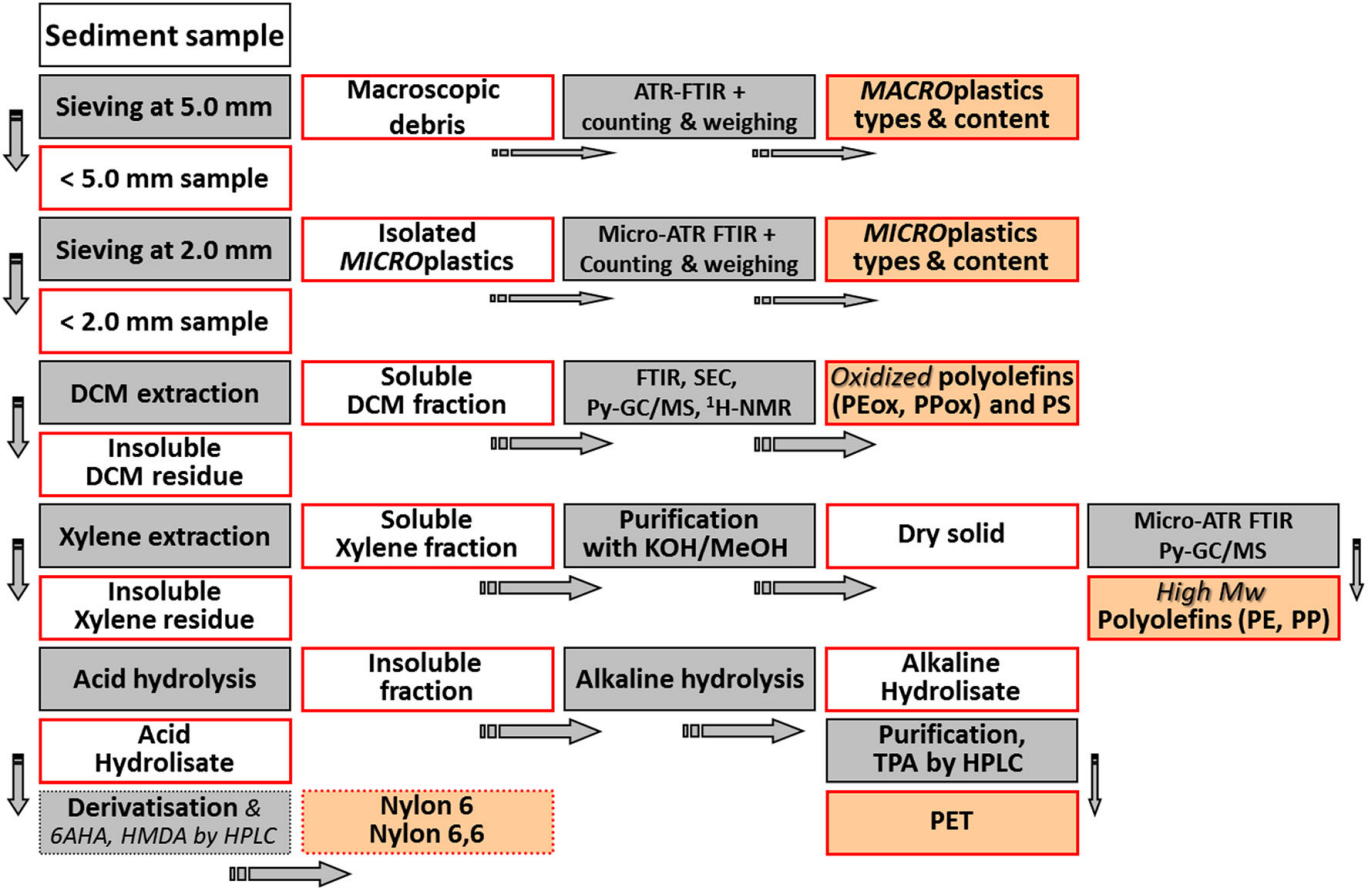
Schematic representation of the experimental protocol for the separation of macroscopic plastics form sediment samples, the subsequent selective extraction and quantification of MPs by polymer types, and the main characterization techniques used for their characterization (DCM, dichloromethane; TPA, terephthalic acid from PET depolymerization; PEox and PPox refer to the oxidized, low molecular weight, DCM-soluble highly degraded polyolefin fraction)

A flowchart depicting the whole analytical procedure for the isolation, identification, and quantification of the individual polymer classes present as larger plastic fragments and, in particular, as MPs in sediment samples is reported in Fig. 3. After the initial sieving to remove and collect the larger plastic debris, the reported procedure comprises dedicated processing routes for the polymers extractable in different solvents (polyolefins, most vinyl polymers including polystyrene, polysiloxanes), and further hydrolytic depolymerization, purification, and quantification steps optimized for the condensation polymers most relevant as MPs in natural water bodies (polyethylene terephthalate, nylons).

In a typical procedure, each sediment sample is pre-sieved at 5 mm (sieve fraction S1), mechanically homogenized, divided into 100–150 g aliquots, and further sieved at 2 mm to obtain the sieve fraction S2, containing plastic fragments with size between 5 and 2 mm, and the passing fraction S3, containing most of the sediment and all MPs smaller than 2 mm. The plastic fragments isolated manually from fractions S1 and S2 are individually weighed on a 0.1 mg precision scale and identified by ATR and micro-ATR-FTIR spectroscopies, respectively. Each one of the type S3 fractions is then sequentially extracted in refluxing dichloromethane (DCM, b.p. 39.6 °C) and then in refluxing xylene (Xy, b.p. 137–144 °C).

DCM effectively extracts PS, low molecular weight (MW), and oxidized polyolefin fragments (e.g., LDPE up to 6–7 kDa), while Xy is effective for high MW LDPE, HDPE, and PP. The DCM extract is then analyzed by gravimetry (solid content) and by SEC with either UV or fluorescence detectors to selectively PS and low MW polyolefins to the content of PS (Biver et al. 2018) and to remove most low MW organic contaminants (among them, phthalates). The Xy fraction is evaporated to a few mL volume and treated with an excess of warm methanolic KOH to precipitate the polyolefins possibly present in the sample and to separate most of the remaining biogenic contaminants soluble either in xylene or in the alkaline aqueous phase. The solid residue from the Xy extraction will contain condensation heteropolymers such as PET and polyamides (nylons) that are insoluble in most organic solvents; these synthetic polymers are then sequentially depolymerized into the corresponding monomers for the subsequent purification and quantitative analysis. For this purpose, in a first step, the residual proteins and synthetic polyamide MPs are depolymerized under acidic conditions. A specific procedure aimed at quantifying the polyamide amino-monomers (e.g., 6-aminocaproic acid from nylon-6 and hexamethylenediamine from nylon-6,6) after derivatization with a fluorophore for the subsequent separation by HPLC and fluorimetric detection has recently been described by Castelvetro et al. (2021). The solid residue from the acid hydrolysis step, mainly consisting of PET and minor residual contaminants, is then treated under strongly alkaline conditions to achieve the complete depolymerization of PET as described by Castelvetro et al. (2020). Briefly, the complete procedure for quantitative analysis of the two polyamides comprises (i) acid depolymerization in aqueous 6 N HCl to quantitatively obtain the monomers 6-aminohexanoic acid (AHA) from nylon-6 and the two comonomers hexamethylenediamine (HMDA) and adipic acid from nylon-6,6; (ii) bulk reaction with dansyl chloride to obtain the dansylated derivatives of HMDA and AHA; and (iii) quantitative determination of AHA and HMDA by reversed-phase HPLC and fluorimetric detection.

Similarly, the complete procedure for quantitative PET analysis comprises (i) alkaline depolymerization in aqueous 1.9 N NaOH to obtain the comonomers terephthalic acid dicarboxylate (or sodium terephthalate, TPA-Na_2_) and ethylene glycol; (ii) purification by solvent extraction and oxidative treatment of the aqueous alkaline hydrolysate; (iii) acidification to convert TPA-Na_2_ into the corresponding dicarboxylic acid (TPA), followed by pre-concentration and further purification of the TPA-containing solution by adsorption/desorption using a reversed-phase cartridge; and (iv) quantitative determination of TPA by reversed-phase HPLC.

The above general protocols have been validated for repeatability, sensitivity, and efficiency of polymer recovery by spiking the environmental samples with known amounts of the polymers of interest (PE, PS, PET, nylon 6, and nylon 6,6).

In addition, detailed information on the composition, structure, degradation level, and VOCs release potential of microplastics and of their fractions obtained through the extraction methods of the validated protocol were obtained by means of a range of hyphenated chromatographic techniques such as Py-GC/MS, SIFT-MS, and NTME-GC/MS.

## Results and discussion

The sediment samples collected in the marine beach (MV) were sieved to separately collect plastic fragments larger than 5 mm and between 2 and 5 mm (sieve fraction S1), respectively, for a first characterization of each individual fragment. The sediment samples sieved at 2 mm mesh from both locations (lakeshore at LB and marine beach at MV) were then analyzed for quantification of the total mass content of each polymer type, according to the protocol depicted in Fig. 3.

### Plastic debris > 5 mm from the MV sediment samples

**Fig. 4:**
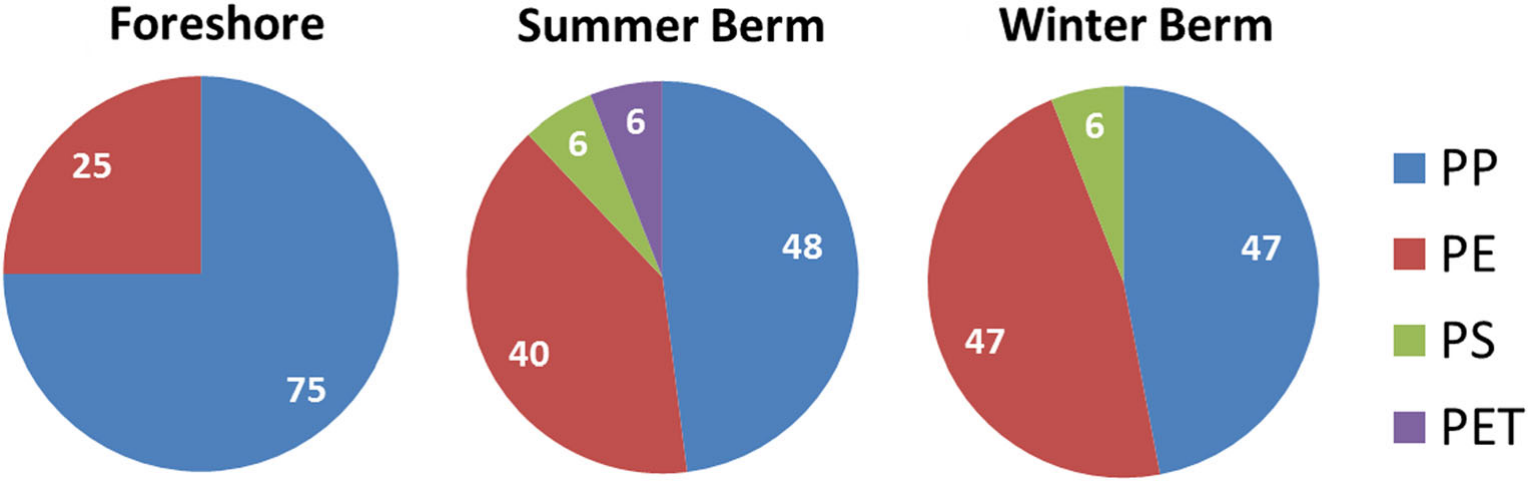
Plastic fragments in MV sampling sectors as identified by ATR-FTIR

Plastic fragments larger than 5 mm (macroplastics) were collected by sieving off from the sediment samples of the MV site. Among the 43 plastic debris collected from the 16 sediment samples of MV, polyolefins (PP and PE) are prevalent in all the shoreline sampling sectors (Fig. 4), with some PS and PET items found in the accumulation zone. They were characterized by recording ATR-FTIR spectra in at least three different spots of each fragment.

**Fig. 5:**
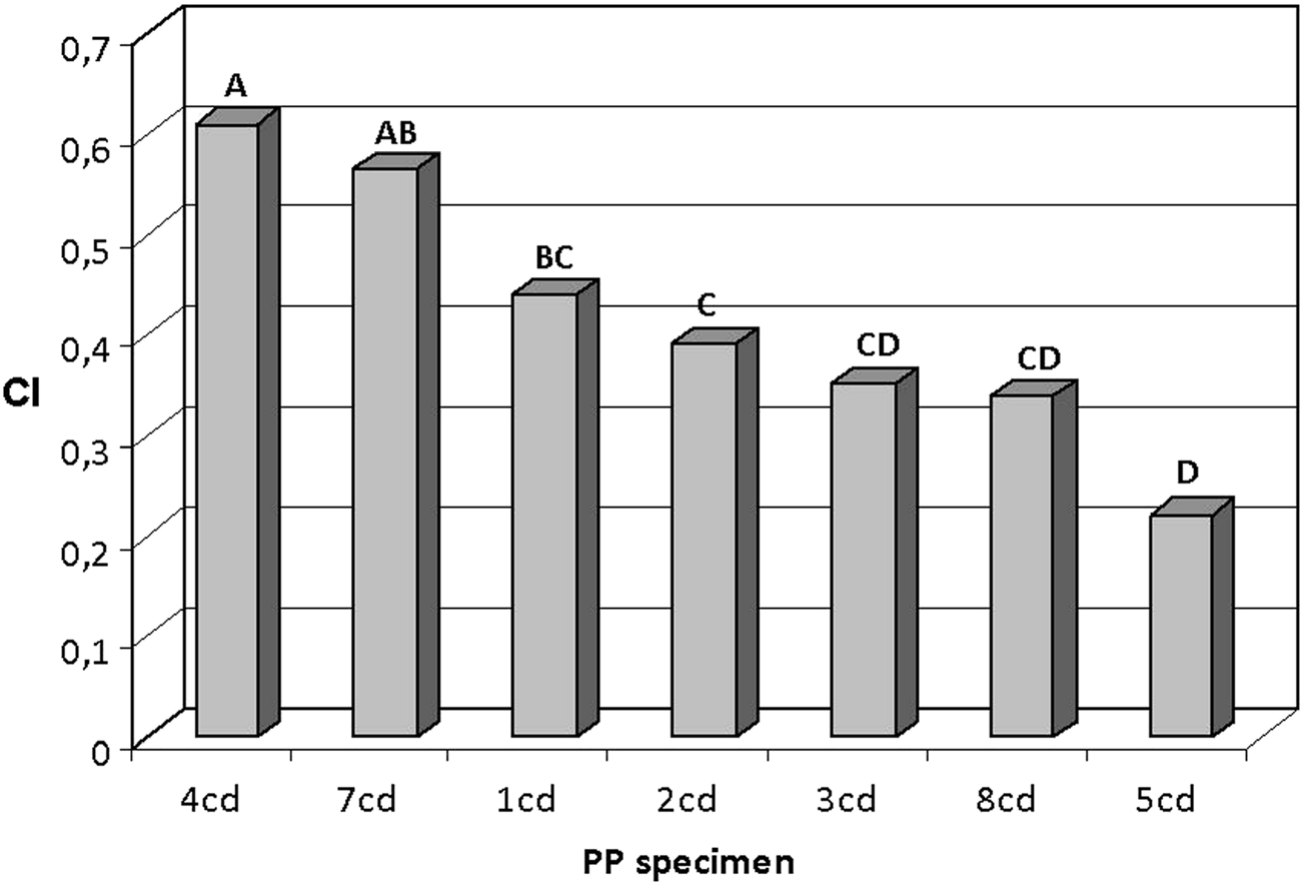
Average CI value for seven PP specimens larger than 5 mm (labeled from 1 cd to 8 cd) sieved off the sediment samples. Each column provides the average CI from three measurements performed in different spots of the specimen to account for the local variability of the oxidation degree. The capital letters (A–D) are indicative of four groups of specimens characterized by statistically dissimilar oxidation degree; two-letter label indicates specimens with features shared by either one of two groups from which they do not show statistically meaningful differences

The presence of absorptions from oxidized functional groups (e.g., hydroxyl O–H stretching absorption around 3400 cm^−1^ and broad absorption from carboxylic, ester, aldehyde, and ketone carbonyl stretching at 1800–1680 cm^−1^) in most of the recorded spectra confirms the expected extensive degradation mainly due to photo-oxidation processes typical of the hydrocarbon polymers. In particular, the structured carbonyl absorption indicates the presence of different functional groups such as aliphatic ketone (1720 cm^−1^), carboxylic acid (1711 cm^−1^), ester (1735 cm^−1^), and lactone (1775 cm^−1^) (Jung et al. 2018; Renner et al. 2017; Yadong et al. 2015). The extent of oxidation has been then evaluated on 7 PP items by calculating the carbonyl index (CI), defined as the ratio of the total area of carbonyl absorption band in the 1810–1680 cm^−1^ range and that of the absorption due to the methylene asymmetric bending vibration in the 1500–1400 cm^−1^ range. For each PP specimen, an average CI value was obtained from the spectra recorded in three different spots. The statistical difference in the degree of oxidation according to the one-way ANOVA carried out with the less significant difference Fisher’s method was also evaluated; the results based on a confidence interval of 95% are summarized in Fig. 5. The different average CI values are indicative of different oxidation levels, and thus of different duration of the environmental exposure for the various PP fragments, suggesting a continuous accumulation of plastic debris in the sampling site. However, heterogeneity in the surface degradation within larger fragments suggests some caution in taking the average CI values alone as representative indicators of the oxidation degree, unless considered along with a statistical analysis of measurements from multiple spots.

### Characterization of MPs > 2 mm

**Fig. 6:**
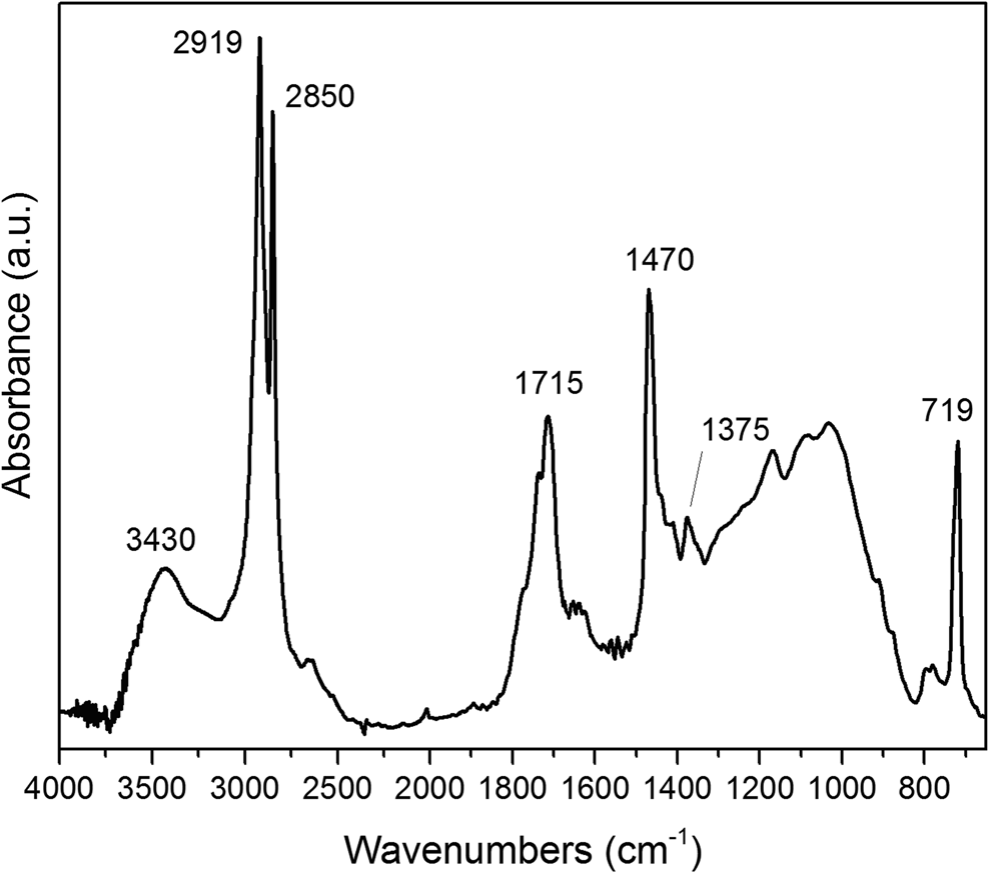
FT-IR spectra of a representative PE fragment (size between 2 and 5 mm) from the MV site. Characteristic LDPE peaks: CH_2_ stretchings at 2919 (asymmetric) and 2850 (symmetric) cm^−1^; CH_2_ scissoring deformation at 1470 cm^−1^; CH_3_ bending (from branching ends in LDPE) at 1375 cm^−1^, and CH_2_ rocking deformation at 719 cm^−1^; hydroxyl (3430 cm^−1^) and carbonyl (1715 cm^−1^) stretching absorptions and broad C–O and similar stretchings in the 1200–1100 cm^−1^

The MPs collected by sieving at 2 mm the sediment samples from the winter berm and dune sectors of the MV site were identified by micro-ATR-FTIR spectroscopy. As shown in Table 1, all MPs were found to be hydrocarbon polymers, with about 1/3 being PE (a representative spectrum is shown in Fig. 6) and the remaining share equally split between PP and PS. Again, the spectral features revealed the presence of a full range of oxidized functional groups (clearly represented by the strong and structured carbonyl absorptions in the 1800–1700 cm^−1^ range and the broad absorption band mainly ascribed to hydroxyl groups centered at about 3430 cm^−1^) as in the larger fragments, suggesting that degradation proceeds with further fragmentation once the plastics waste reaches the accumulation zones in the shoreline sectors, in agreement with previous observations (Corcoran et al. 2009, Castelvetro et al. 2018). It is worth noting that the level of oxidative degradation was comparable to that reported in the case of artificially aged PE and PS samples (Lacoste et al. 1993; Gardette et al. 2013; Emad and Haddad 2013).

**Table 1:** Micro-ATR-FTIR identification of MPs collected from shoreline sediment samples of MV

Sampling sector	Sediment sample	Plastic fragment	Weight (mg)	Polymer
Winter berm	MV-05	f	37.0	PE
		g	27.8	PE
	MV-06	f	31.5	PE
		h	10.2	PS
		i	12.3	PE
	MV-07	r	44.4	PP
		s	10.4	PE
		t	59.3	PS
		u	10.1	PE
		v	12.7	PE
	MV-08	f	37.3	PE
		g	21.8	PE
		h	12.8	PP
Dunes	MV-03	a	26.4	PS
	MV-04	c	28.5	PE
		D	30.6	PP

Another significant feature was the different numerical distribution of polymer types when comparing the plastic fragments recovered from the sieve fractions S1 and S2 of the MV samples. In particular, PP was found as the most common plastic debris in fraction S1 (> 5 mm), whereas PE MPs were dominant in fraction S2 (2 < size < 5 mm); this is likely to be not incidental, but rather a consequence of the different propensity to fragmentation of PP and PE, the former being characterized by a higher tendency to undergo surface fragmentation directly intopowdery material that is hardly recognizable even by microscopy-assisted manual sorting.

**Fig. 7:**
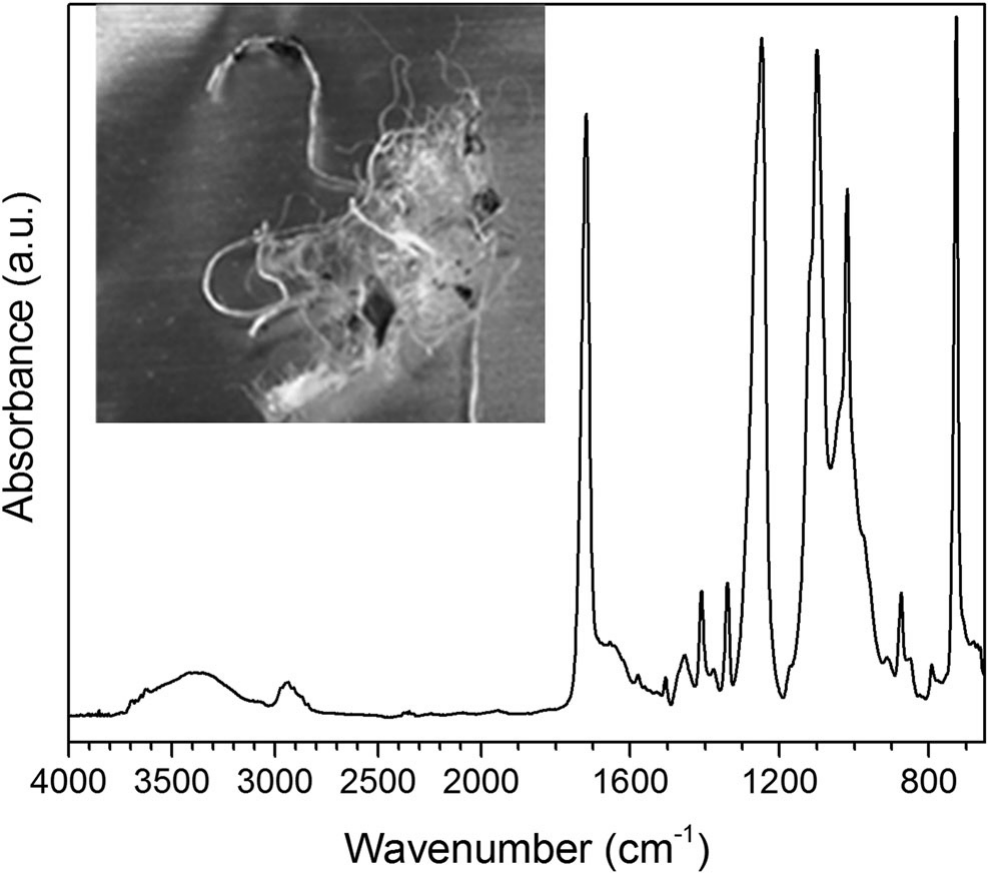
Micro-ATR-FTIR spectrum of fragment LB2-B2-F1 (in the inset the optical microscopy image of the corresponding microfiber bundle)

The LB sediment samples had been already sieved on-site prior to the delivery for laboratory analysis; therefore, no fragments larger than 5 mm were left. Here the results of the analyses carried out on only a selected set of samples are discussed for comparative purposes, as a more detailed report can be found in the recent paper by Corti et al. (2020). In particular, samples representative of both LB sites (LB1 and LB2) and of the horizons B and C, farther apart from the shoreline and thus less heavily influenced by erratic action of surf water from the wave action, are considered. From the micro-ATR-FTIR analysis of the MPs larger than 2 mm, as listed in Table 2, five types of synthetic polymers could be detected: in addition to the usual LDPE, PP, and PS, traces of acrylic fibers (acrylonitrile copolymer, AN) and a prevailing presence (36%) of PET mainly in the form of synthetic polyester fibers were identified in the sieve fraction. Similarly to the plastic fragments from the MV site, the polyolefin fragments from LB showed high levels of oxidation (intense IR absorptions from hydroxyl and aliphatic carbonyl groups). The micro-ATR-FTIR spectrum of a representative PET fragment is shown in Fig. 7.

**Table 2:** Micro-ATR-FTIR identification of MPs collected from LB sediment samples (Corti et al. 2020)

Sampling site	Sample	Macroplastic fragment	Weight (mg)	Polymer
LB1	LB1-B3	F1	40.5	LDPE
		F2	8.9	PP
	LB1-C3	F1	1.6	PP
		F2	0.4	PS
		F3	0.6	PS
		F4	n.d.	PET + AN
LB2	LB2-B2	F1	1.2	PET
		F2	0.5	LDPE
	LB2-C2	F1	0.6	PET
		F2	23.9	PET
		F3	24.3	PP

The relative abundance of the different polymer types recovered as fragments larger than 2 mm from the sediment samples marked a clear difference between the two sites: while only hydrocarbon polymers (PE, PP, and PS) were identified in the samples from MV, those from LB included also heteropolymers such as PET. Such difference, although based on a small number of collected fragments and thus with limited statistical validity, is clearly related to the specific features of the sampling sites. In particular, the contamination of the MV site, a marine coastal beach, is likely to be mainly due to less dense polymers eventually stranded after their journey as floating plastic debris. On the other hand, the sampling site in LB was expected to represent a mixed condition comprising both the shore and the lakebed accumulation, as the sediment samples were collected near the shore but during a drought period that had temporarily left dry an area previously submerged by the lake waters. The drought period may have uncovered sediments contaminated also by high density debris such as those consisting in the heteropolymer PET.

### DCM-soluble polymer fraction

The extraction of fraction S3 (< 2 mm) of the sediments with refluxing dichloromethane allows recovery of PS and of the most heavily oxidized and chain-fragmented fraction of polyolefins; in addition, other vinyl polymers (e.g., acrylics, polyvinyl chloride) less frequently found in beach sediments and uncrosslinked silicones (polydimethylsiloxane) could also be present. All the dichloromethane extracts were analyzed by means of spectroscopic (FT-IR) and chromatographic (SEC, Py-GC/MS) techniques.

**Fig. 8:**
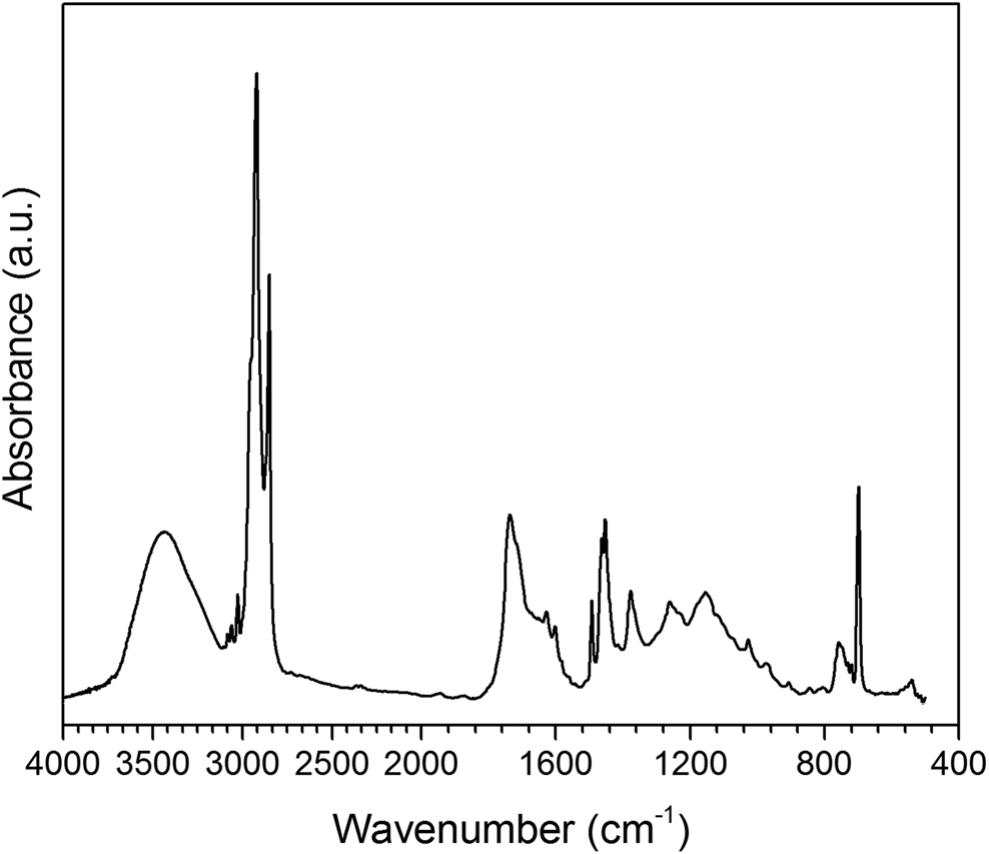
FT-IR spectrum of the DCM extract of MV-07 sediment sample from the winter berm sector

In Fig. 8 the FTIR spectrum recorded from a representative sediment DCM extract (from sample MV-07) shows the absorption bands indicative of a mixture of polyolefins, with traces of polystyrene. In particular, the presence of possibly both HDPE and LDPE is highlighted by the strong methylene C–H stretching (at 2917 and 2848 cm^−1^) and bending (at 1453 cm^−1^) absorptions as well as methyl C–H stretching (small shoulder at 2850 cm^−1^) and bending (at 1377 cm^−1^) absorptions, the intensity of the latter suggesting that PP represents a significant fraction of the extract. Besides, the intense absorptions in the carbonyl regions (1700–1800 cm^−1^) and the broad absorptions in the C–O stretching regions (1000–1300 cm^−1^) along with the absorptions related to the presence of O–H groups (hydroxyl, carboxylic acid, and possibly from some absorbed water) in the 3300–3600 and 1600–1700 cm^−1^ are indicative of a high level of oxidation of these polyolefins. In addition, the small but well-defined aromatic C–H stretching absorptions at 3000–3050 cm^−1^ and the sharp peak from the out-of-plane aromatic C–H deformation at 690 cm^−1^ indicate a not negligible amount of PS. The Py-GC/MS analysis performed on the same DCM extract from MV-07 featured oligomeric linear hydrocarbon fractions ranging between 7 and 36 carbon atoms (from n-heptane to n-hexatriacontane and relevant mono and di-unsaturated homologs). This profile is consistent with the pattern obtained by analyzing a sample of reference oxidized LDPE obtained by thermal treatment of an oxo-biodegradable LDPE produced in our laboratory. Analogous results were obtained for the DCM extracts of most of the MV sediment samples, with only a few samples presenting detectable chromatographic peaks deriving from the pyrolytic depolymerization of PS, identified from the ionic fragments at m/z 104 (styrene molecular ion) and 91 (tolyl ion, typical of styrene decomposition pattern).

The FT-IR spectra recorded from the DCM extracts of the LB sediment samples showed the same general features already observed in those from the MV site, with one noteworthy difference consisting in the presence of additional absorptions typical of silicones (polydimethylsiloxane and its parent species) at 1261 cm^−1^ (C–Si–C in-plane scissoring), 1095 and 1022 cm^−1^ (Si–O–Si symmetric stretching), and 804 cm^−1^ (out-of-plane CH_3_ bending and Si–C symmetric stretching). The presence of silicones in freshwater systems may be associated to the pollution from domestic and industrial wastewaters as silicones are often used in the formulations of personal care, household, and industrial products.

While the total content of synthetic polymers in the DCM extracts can be determined by gravimetry, for a more sensitive and polymer-specific determination of the PS fraction, we adopted a semi-quantitative analytical procedure based on chromatographic analysis. The separation was performed by SEC, and the high MW fraction (retention time around 10 min) was quantified by UV (DAD) and/or fluorescence (FLD) detectors according to a calibration curve built by analyzing standard PS solutions in the appropriate concentration range. The procedure, already described by Biver et al. (2018), allows a fairly accurate quantification of PS in the high MW range (the main source of error being associated with possible differences in the absorption coefficient and fluorescent emission of oxidized vs. non-aged PS at the set wavelengths of the DAD and FLD detectors, respectively); such high MW fraction consists of nearly pure PS since polyolefins, even if partially oxidized, are only extracted by DCM if their MW does not exceed few thousand Daltons. Besides, the wavelength for detection can be set as to avoid detection of other high MW vinyl polymers such as e.g. acrylics (although their presence is generally negligible). A high MW fraction was found in the DCM extracts from MV samples farthest from the shoreline and in one of the LB samples, as reported in Table 3. In addition to the high MW fraction, all SEC profiles of the DCM extracts for both MV and LB sediments showed a well-resolved low MW fraction eluted at high retention times (15–17 min), mainly consisting of mixtures of highly oxidized polyolefin and PS oligomers.

**Table 3:** High molecular weight PS content in selected DCM extracts from MV and LB, as determined by SEC with DAD calibrated detector

MV site	Sample	PS (mg/kg)	LB site	Sample	PS (mg/kg)
Summer berm	MV-12	0.15	LB1	LB1-A1	0.22
Foreshore	MV-15	0.06		LB1-C2	0.09
Winter berm	MV-05	1.06		LB1-B3	0.08
	MV-06	0.39		LB1-C3	0.95
	MV-08	1.08	LB2	LB2-A2	1.54
Dunes	MV-01	1.49		LB2-C2	0.19
	MV-02	3.67		LB2-A3	0.11
	MV-04	24.92 ^a^		LB2-C3	0.02

The results in Table 3 are based on a number of subsamples too limited for the variability expected in natural shore sediments to either confirm or deny any statistically significant difference of PS MPs concentration in the two sites. Nevertheless, a consistently higher level of contamination in the shore sediment of MV than in the mixed sediment of LB seems to emerge from these data; if confirmed, this would be in agreement with a preferential deposition of floating PS ashore rather than in bottom sediments. On the other hand, the data for the MV samples show a statistically significant higher variability than the LB ones (F test, 95% confidence level), with increasing content of PS moving away from the shoreline towards the dune sector where plastic fragments transported by waves and dominant winds are more easily accumulated, undergoing long-term degradation into MPs eventually buried in the sandy sediment. The lower variability among the LB samples is likely a result of the fluctuation of the shoreline caused by the seasonal variation of the water level.

### Polymer fraction extracted in refluxing xylene

As a second step of the analytical protocol, the residues from the DCM extraction are further extracted with refluxing xylene. For the LB samples, containing a larger fraction of biogenic material compared to the MV ones, the standard procedure described by Ceccarini et al. (2018) was slightly modified to improve the purification and recovery yield of the extracted high molecular weight polyolefins. In particular, the obtained xylene extract solutions were concentrated from the initial 200 mL to about 20 mL by distilling off the excess xylene; then 30–50 mL methanol was added to precipitate the polymeric fraction that was easily recovered by filtration on 0.22 μm PVDF filter (caution is required to avoid PVDF filters with LDPE support). After drying and weighing, only a few mg of solids could be recovered from the xylene extract of each analyzed LB and MV sample, corresponding at most to a few ppm of mildly oxidized polyolefins, as identified by micro-ATR-FTIR directly on the PVDF filter membrane. Due to the very limited number of observations, these results cannot provide an accurate picture of the actual content of high MW polyolefin MPs in the sediments of the two sites. Nevertheless, some general information could be drawn from the observation of the site-dependent MPs concentration, polymer composition, and extent of polymer degradation found through the above discussed simple solvent extraction and analysis procedures. In particular, polyolefin MPs are mainly found as DCM-extractable, heavily oxidized low MW fractions throughout the MV sampling sites, while much lower concentrations of polyolefin MPs were found in the sediment of mixed origin (bottom sediment with recent conversion into a shore one) sampled in LB. This is in agreement with the well-known sensitivity of polyolefins to the photo-oxidative stress boosted by environmental exposure to high solar irradiation and temperatures as typically found in sandy beaches during the summer season, as opposed to the deposition in lakebed sediments. Finally the less uniform distribution found for the PS MPs in the MV marine beach, with higher concentration in the accumulation zones farthest from the shoreline, may be the result of a degradation pattern for PS different from that of polyolefins, with preferential formation of short and oxidized fragments that can be more easily removed as soluble oligomers or colloidal particles from the sandy sediment under the surf action of the waves.

### Analysis of the total PET content in the sediment samples

To quantify the PET content in the sediment samples, the depolymerization procedure described by Castelvetro et al. (2020) was adopted. The analyses were performed on the residues from the sequential extractions with DCM and xylene, from which all polyolefins and vinyl polymers as well as other potential interferents in the analysis of TPA such as low MW phthalates (nearly ubiquitous environmental contaminants) had been removed.

Three representative sediment samples collected from different zones of the MV site (foreshore, backshore winter berm, dunes) and all the sediment samples from LB were treated with hot aqueous NaOH 1.9 M in the presence of a phase transfer catalyst, and the resulting hydrolysates were purified according to the reported procedure to remove most of the TPA contaminants before reversed-phase HPLC analysis. The results listed in Table 4 are obtained from at least three replicates (subsamples) of each sample. The large variability within the same sample, as indicated by the large confidence interval, is likely to be the result of persistent heterogeneity within the same natural sediment sample even after mechanical homogenization, rather than to the precision of the method as the latter had been previously validated (Castelvetro et al. 2020).

**Table 4:** Analysis of PET content in subsamples of MV and LB sediments

Sampling area	Sample	PET^a^ (mg/kg)	Confidence interval^b^	Sampling site	Sample	PET^a^ (mg/kg)	Confidence interval^b^
Foreshore	MV-16	0.37	0.09	LB1	LB1-H1^c^	3.83	0.60
					LB1-H2^c^	3.44	0.31
Winter berm	MV-08	0.43	0.03		LB1-H3^c^	3.97	0.08
				LB2	LB2-A2	2.92	0.99
Dunes	MV-04	0.46	0.03		LB2-B2	9.87	3.05
					LB2-C2	36.81^d^	6.61
					LB2-A3	0.82	0.27
					LB2-B3	0.93	0.59
					LB2-C3	4.17	6.09
Average		0.41 ± 0.07		Average		3.74 ± 2.80	

^a^mg PET/kg sediment, calculated from the TPA content determined by reversed-phase HPLC

^b^Confidence level = 95%, sample size = 3

^c^H1, H2, and H3 indicate samples obtained by combining the extraction residues from all three samples of the same horizon

^d^Outlier

As expected, a statistically significant higher contamination level was found in the LB sediments, as they are of mixed (bottom-shore) type and are thus more likely to become a sink for high density MPs such as those consisting of PET and other heteroatom-containing polymers. On the other hand, the not negligible PET content in the MV beach sediments, although based on a limited and number of analyzed samples, indicates that MPs of this high density polymer, likely consisting of microfibers originally released in urban wastewaters (Napper and Thompson 2016; Belzagui et al. 2019; Gatidou et al. 2019), can be transported at fairly long distance by marine currents and turbulent surface waters, eventually ending up not only in the bottom sediments but also in coastal ones. While a minor contribution to the sediment contamination from airborne PET MPs cannot be excluded, the fragmentation mechanism actively contributing to the MPs generation from larger polyolefin items is very unlikely to contribute to the generation of PET secondary MPs since both photo-oxidative and hydrolytic PET degradation mechanisms are more likely to result in the release of soluble molecular or oligomeric fragments.

### Microplastics photodegradation under simulated environmental aging

Artificial photo-aging was performed on a set of reference micronized polymers (LLDPE and HDPE polyethylenes, polypropylene, polystyrene, and polyethylene terephthalate), hereafter reference MPs, using a Solar Box aging chamber. The selected polymers are those most commonly found as microplastics in water bodies. The effects of photo-oxidative degradation on the polymer structure after 1, 2, 3, and 4 weeks of Solar Box exposure were evaluated using analytical pyrolysis-gas chromatography/mass spectrometry (Py-GC/MS), while the VOCs released as a result of photo-oxidation were captured and analyzed with two distinct techniques: headspace (HS) with needle trap microextraction (NTME) combined with gas chromatography/mass spectrometry (GC/MS) analysis, as described by Lomonaco et al. (2020), and selected ion flow tube-mass spectrometry (SIFT-MS), as described by La Nasa et al. (2021). Some of the Py-GC/MS and SIFT results are presented and discussed below.

#### HDPE and LDPE

**Fig. 9:**
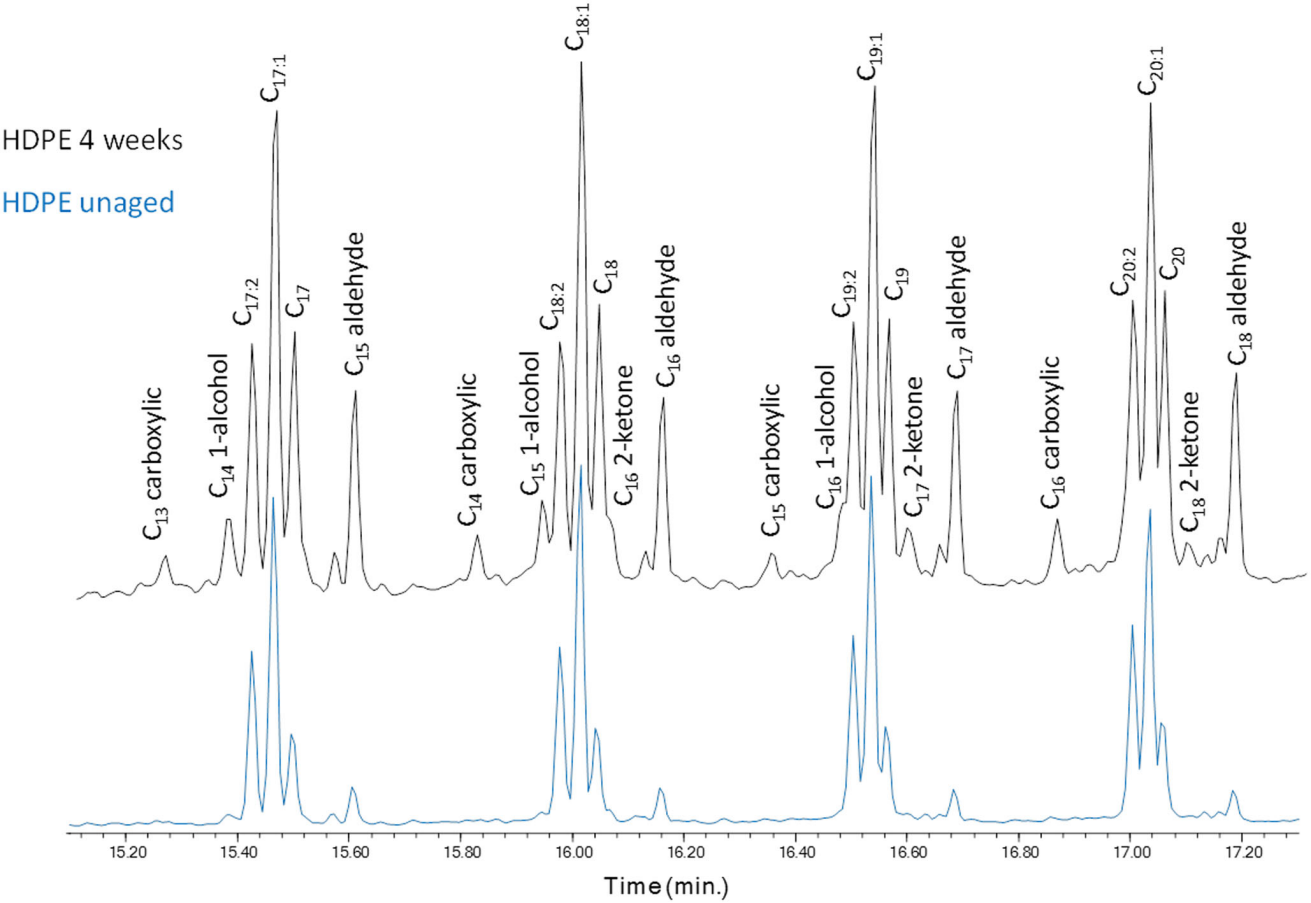
Comparison of the Py-GC/MS profiles of unaged and artificially aged HDPE

The pyrolytic (Py-GC/MS) profiles of the virgin HDPE and LDPE were similar. The most relevant feature in all chromatograms was the presence of a series of clusters comprising three main peaks each, assigned to the diene (most likely an α,ω-diene), the monoalkene (most likely a 1-alkene), and the alkane, respectively, of a given C_n_ hydrocarbon. These clusters were characterized by chain lengths in the range C_2_–C_34_ for both polyethylenes. After artificial aging, the appearance of new species next to the triplets was observed in the profiles of both polymers, associated with the formation of linear ketones, monounsaturated and saturated aldehydes, and monocarboxylic acids (Fig. 9), with lengths up to C_24_. The complete lists of all the pyrolysis products detected in the pyrograms of the two polyolefins before and after artificial aging are reported in Tables S1-S2 in the *Online Resource*.

While the general features of the pyrograms from the two aged polymers were similar, in the case of HDPE, a higher number of oxidized pyrolysis products were recorded compared to LDPE. Due to the drastic conditions of the pyrolytic treatment, the detected oxidation products could either result from the thermolytic cleavage of oxidized high MW polymer chains, or from the thermal desorption of smaller oxidized fragments present in the bulk polymer particle as a result of photo-oxidation, or both.

**Fig. 10:**
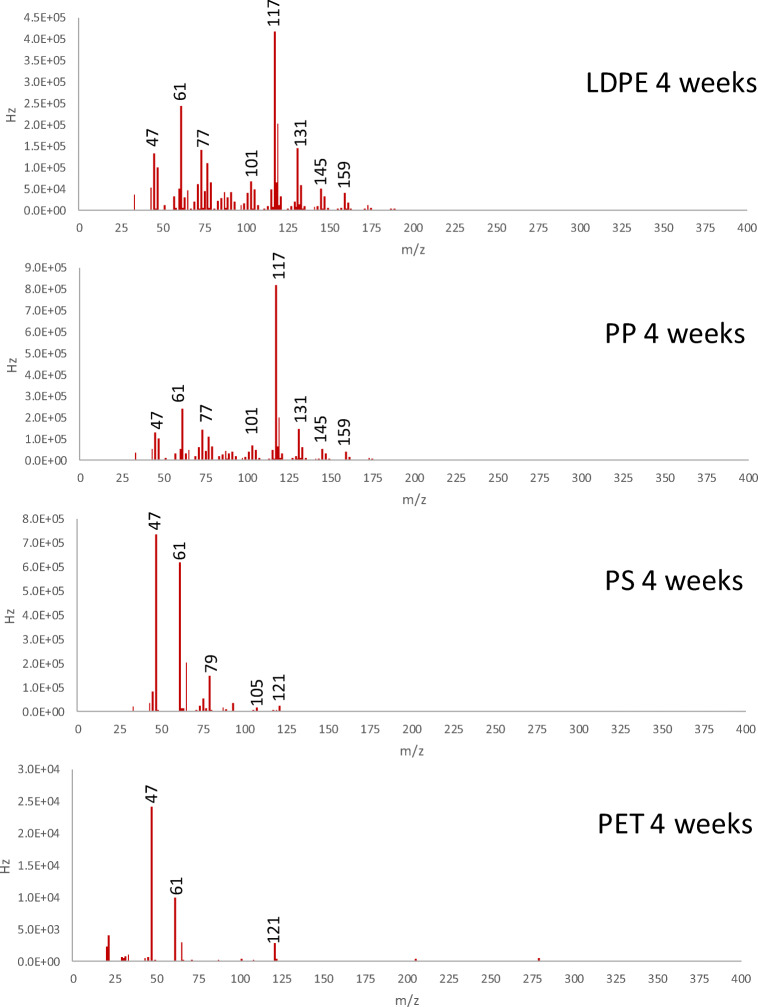
Artificially aged MPs: SIFT-MS mass spectra obtained with [H_3_O]^+^ reactive ion

The SIFT-MS spectra obtained from the reference polyolefins after artificial aging (in Fig. 10 are shown the MS spectra from all 4 reference polymers obtained with [H_3_O]^+^ as the reactive ion) were characterized by the presence of a relative high abundance of acetone (m/z = 77 and 107) as well as smaller amounts of aldehydes, alcohols, and carboxylic acids in the C_2_–C_12_ range. These results, complementary and in agreement with those obtained by Py-GC/MS, suggest that the aldehydes detected in the pyrolysis profile were not only the result of pyrolytic chain fragmentation of oxidized polymer chains but were also present as low MW photodegradation by-products released by desorption from the bulk polymer at moderate temperature.

#### PP

The pyrolytic profile of polypropylene features 2,4-dimethyl-1-heptene as the main pyrolysis product, together with PP oligomers deriving from pyrolytic chain scissions. The complete lists of all the pyrolysis products detected in pristine and aged PP are reported in Tables S3-S4 in the *Online Resource*.

Interestingly, the pyrolytic profile recorded after 4 weeks of artificial aging did not present any significant difference from that of the unaged polymer. A detailed analysis of the pyrogram obtained by selecting the response to the characteristic ions of aldehydes highlighted traces of low molecular weight species (up to C_12_).

The results of the SIFT-MS analysis were similar to those obtained for HDPE and LDPE (Fig. 10). The most relevant features in the VOCs composition were the presence of aldehydes and carboxylic acid in the range C_2_–C_12_, together with a predominance of acetone. Alcohols up to C_6_ were detected in traces. The results of the VOC analysis of these materials, as in the case of the two polyethylenes, suggest that the aldehydes detected by the Py-GC/MS analysis are not only resulting from the pyrolysis of the polymer but have also formed during the artificial aging.

#### PS

The pyrolytic profile of PS featured styrene and its dimers and trimers as the main products. The analytical pyrolysis performed after artificial aging showed only a slight variation of the relative abundances of the pyrolysis products (styrene vs. oligomers). This behavior agrees with the typical degradation processes of PS, characterized mainly by depolymerization pathways. The complete lists of all the identified species in virgin and aged polystyrene are reported in Tables S5-S6 in the *Online Resource*.

The SIFT-MS spectrum of the aged polymer was significantly different from those obtained from the polyolefins (Fig. 10). The most abundant species were acetone, together with formic and acetic acids. Moreover, the SIFT-MS analyses also detected aromatic compounds with benzene, styrene, styrene oxide, and benzaldehyde as most abundant.

#### PET

The pyrolytic profile of virgin PET was mainly characterized by the presence of benzoic acid, vinyl benzoate, divinyl benzoate, and their relative dimers and trimers. Several oxidation products could be detected after artificial aging, with acetophenone, benzaldehyde, vinyl benzoate, dibenzofuran, and fluorenone as the most abundant. The complete lists of all the species detected in the Py-GC/MS profiles of fresh and aged PET are reported in Tables S7-S8 in the *Online Resource*.

The SIFT-MS spectra of the aged PS were less rich than those of all other polymers (Fig. 10). In particular, the profile after 4 weeks of aging was mainly characterized by the presence of the same oxidation products as those detected by Py-GC/MS, acetophenone and benzaldehyde as the most abundant species.

### Discussion on the qualitative and quantitative profile of the released VOCs in relation with the extractable fraction of oxidized degradation products of photo-aged microplastics

**Fig. 11:**
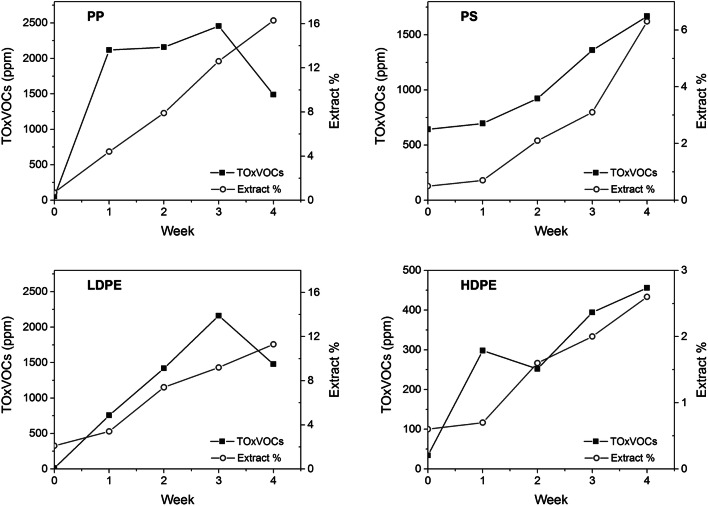
Total oxidized VOCs (TOxVOCs, expressed as μg VOCs/g polymer) emitted by the four hydrocarbon polymers over 4 weeks of photo-aging, in relation with the extractable fraction

The progress of degradation in the photo-aged micronized polymers was also evaluated by periodically checking the amount of oligomeric and/or oxidized fraction extractable in DCM (for polyolefins and PET) or methanol (for PS) with parallel qualitative and quantitative evaluation of the total amount of volatile oxygenated species (TOxVOCs, i.e., ketones, lactones, esters, carboxylic acids, aldehydes, alcohols, and ethers) by NTME-GC/MS, as previously reported by Lomonaco et al. (2020). The lowest release was recorded for PET followed by PS, as a consequence of their higher stability against photo-oxidation due to the partially aromatic structure and, in the case of PET, to the absence of labile hydrogen atoms on tertiary carbons. Figure 11 summarizes the results concerning the four hydrocarbon polymers, that is, those representing the largest fraction of plastic waste polluting the marine environment and also those more sensitive and more likely to be exposed to photo- and thermo-oxidative environmental stresses because of their low density. A good correlation is apparent between the nearly linear growth of solvent-extractable fraction and of TOxVOCs, respectively, for all polymers. The only exception is a TOxVOCs reduction for the most sensitive (because highly branched and thus with higher density of labile tertiary C–H bonds) PP and LDPE at the 4th week of aging. Such apparently surprising behavior may be explained by an enhanced effectiveness of the increasingly oxidized polyolefins as adsorbers of low MW polar compounds, with a consequent depletion of the amount of oxygenated VOCs in the gaseous phase. Such general behavior had been described by Chiellini et al. (2006) in an investigation on the degradation behavior of polyolefins added with transition metal catalysts promoting thermodegradation by a similar free radical mechanism.

## Conclusions

The described multi-step fractionation and purification protocol and multianalytical approach allowed the accurate polymer-specific detection and quantification of the total mass content of contaminating MPs and NPs (although NPs are unlikely to significantly contribute to the overall mass if MPs are present) in complex matrices such as marine and lakebed coastal sediments. The bulk analysis approach described in this paper should be considered complementary to the conventional one based on particle isolation and counting as it provides more accurate quantitative results and new insights on the effects of the environmental degradation of MPs, but involves loss of information on the size, shape, color, and extent of degradation of the individual particles.

In the case of hydrocarbon polymers such as PP, PE, and PS, solvent extraction followed by chromatographic (SEC) separation allowed PS detection in the range from 0.06 to 25 ppm in the sandy marine beach sediments. Polyolefins were mostly present as highly oxidized low MW fragments, uniformly distributed throughout the beach sectors. Quantification of PET MPs down to a few tens of ppb was made possible by the recently devised procedure based on PET depolymerization and quantitative analysis of the TPA comonomer. A recently devised extension of the protocol adopted in this work, which includesthe detection and quantification of nylon 6 and nylon 6,6 polyamides, allows to further improve the thoroughness and usefulness of this methodology in environmental studies. Finally, the broad range of VOCs released at increasing rates by MPs as their photo-oxidative degradation proceeds, particularly in the case of polyolefins and polystyrene (arguably the most abundant MPs to be found on exposed coastal sediments), provides a clear evidence that MPs are a not negligible source of pollution of both water and atmosphere, in addition to posing potential threaths to living organisms due to the toxicity of some of the released VOCs. On the other hand, the same results highlight that the exposure of MPs to environmental photo-oxidative and thermal stress is likely to result in shorter than anticipated persistence of these materials in the environment.

## Supplementary information

Supplementary Material . The following supplementary material is available in the online version: Tables S1-S8 collect the species detected in the Py-GC/MS profiles of the pristine and aged reference polymers. (DOCX 148 kb)
