# Supplementary material for: New methodologies for the detection, identification, and quantification of microplastics and their environmental degradation by-products
Source: Environ Sci Pollut Res Int. 2021 Jan 27;28(34):46764–80. doi: 10.1007/s11356-021-12466-z (PMC8384832; doi:10.1007/s11356-021-12466-z)
Supplement: Supplementary file 1 — . The following supplementary material is available in the online version: Tables S1-S8 collect the species detected in the Py-GC/MS profiles of the pristine and aged reference polymers. (DOCX 148 kb) [file 11356_2021_12466_MOESM1_ESM.docx]

**Environmental Science and Pollution Research**

**Online Resource 1**

**New methodologies for the detection, identification, and quantification of microplastics and their environmental degradation by-products**

Valter Castelvetro^1,2,*^, Andrea Corti^1^, Greta Biale^1^, Alessio Ceccarini^1^, Ilaria Degano^1,2^, Jacopo La Nasa^1^, Tommaso Lomonaco^1,2^, Antonella Manariti^1^, Enrico Manco^1^, Francesca Modugno^1,2^, Virginia Vinciguerra^1^

^1^ Department of Chemistry and Industrial Chemistry, University of Pisa, 56124 Pisa, Italy

^2^ CISUP - Center for the Integration of Scientific Instruments of the University of Pisa, University of Pisa, 56124 Pisa, Italy

* [*valter.castelvetro@unipi.it*](mailto:valter.castelvetro@unipi.it)

In Figure S1-S2 are reported the FT-IR spectra of representative DCM extracts from sediment samples of both sampling sites (MV and LB), showing diagnostic absorptions for the given prevalent synthetic polymer (Jung 2018; Renner 2017)^[[1]](#footnote-1)^.

The FT-IR spectrum of the extract from aged LB1-A3 (Figure S1) shows absorption bands at 2917, 2848, 1453, and 1375 cm^-1^ from linear aliphatic chains with some branching. The structured weak carbonyl stretching band at 1800-1700 cm^-1^ indicates a low concentration of oxygen-containing moieties from the oxidation of the polyolefin (possibly a mixture of HDPE, LDPE and PP).





**Fig. S1** FT-IR spectrum of the DCM extract of LB1-A3 sediment sample





**Fig. S2** FT-IR spectrum of the DCM extract of MV-02 sediment sample

The FT-IR spectrum of the DCM extract of the aged MV-01 (Figure S2) allows to identify a either predominant or exclusive content of PS (aromatic absorption bands from C-H stretching at 3050 and 3025 cm^-1^; overtones detectable between 2000 and 1800 cm^-1^; out of plane aromatic C-H deformation at 700 cm^-1^. This was confirmed by Py-GC/MS analysis, in which a GC peak at 7.02 min retention time which was characterized for the presence of a ionic fragment having 104 m/z corresponding to the PS molecular ion (M+) accompanied by 91 m/z peaks relevant to the styrene and styrene oligomers decomposition pattern.

**Table S.1 –** Pyrolysis products identified by Py-GC/MS for unaged HDPE

| # | t_r_ | Peak identification | Main ions (m/z) |
| --- | --- | --- | --- |
| 1 | 2.7 | 1-hexene | 84, 69, 56, **41** |
| 2 | 4.0 | 1-heptene | 98, 83, 70, **56**, 41 |
| 3 | 4.1 | heptane | 100, 71, 57, **43** |
| 4 | 6.8 | 1-octene | 112, 97, 83, 70, **55**, 41 |
| 5 | 7.0 | octane | 114, 85, 71, 57, **43** |
| 6 | 8.7 | 1,8-nonadiene | 109, 96, 81, **67**, 55, 41 |
| 7 | 8.9 | 1-nonene | 126, 97, 83, 69, **56**, 41 |
| 8 | 9.0 | nonane | 128, 99, 85, 71, 57, **43** |
| 9 | 10.2 | 1,9-decadiene | 110, 95, 81, 67, **55**, 41 |
| 10 | 10.3 | 1-decene | 140, 111, 97, 83, 70, 55, **41** |
| 11 | 10.4 | decane | 142, 117, 99, 85, 71, **57**, 43 |
| 12 | 11.2 | 1,10-undecadiene | 124, 109, 95, 81, 67, 55, **41** |
| 13 | 11.3 | 1-undecene | 154, 126, 111, 97, 83, 70, 55, **43** |
| 14 | 11.4 | undecane | 156, 98, 85, 71, **57**, 43 |
| 16 | 12.1 | 1,11-dodecadiene | 138, 124, 109, 95, 81, 67, **55**, 41 |
| 17 | 12.16 | 1-dodecene | 168, 140, 125, 111, 97, 83, 69, 55, **41** |
| 18 | 12.2 | dodecane | 170, 128, 85, 71, **57**, 43 |
| 19 | 12.3 | decanal | 138, 128, 112, 95, 82, 68, 55, **41** |
| 21 | 12.87 | 1,12-tridecadiene | 123, 109, 95, 81, 67, **55**, 41 |
| 22 | 12.9 | 1-tridecene | 125, 111, 97, 83, 69, **55**, 41 |
| 23 | 13.0 | tridecane | 184, 99, 85, 71, **57**, 43 |
| 24 | 13.05 | undecanal | 152, 126, 109, 96, 82, 68, **57**, 41 |
| 27 | 13.5 | 1,13-tetradecadiene | 123, 109, 96, 81, 67, **55**, 41 |
| 28 | 13.6 | 1-tetradecene | 125, 111, 97, 83, 69, **55**, 41 |
| 29 | 13.7 | tetradecane | 198, 99, 85, 71, **57**, 43 |
| 30 | 13.75 | dodecanal | 166, 140, 123, 110, 96, 82, 68, **57**, 41 |
| 33 | 14.2 | 1,14-pentadecadiene | 123, 109, 96, 81, 67, **55**, 41 |
| 34 | 14.27 | 1-pentadecene | 210, 125, 111, 97, 83, 69, **55**, 41 |
| 35 | 14.3 | pentadecane | 212, 113, 99, 85, 71, **57**, 43 |
| 36 | 14.4 | tridecanal | 180, 154, 124, 110, 96, 82, 68, **57**, 43 |
| 39 | 14.8 | 1,15-hexadecadiene | 123, 109, 96, 82, 67, **55**, 41 |
| 40 | 14.88 | 1-hexadecene | 224, 125, 111, 97, 83, 69, 55, **41** |
| 41 | 14.9 | hexadecane | 226, 99, 85, 71, **57**, 43 |
| 42 | 15.0 | tetradecanal | 194, 168, 138, 110, 96, 82, 69, **57**, 41 |
| 45 | 15.4 | 1,16-heptadecadiene | 137, 123, 109, 96, 82, 69, **55**, 41 |
| 46 | 15.46 | 1-heptadecene | 238, 139, 125, 111, 97, 83, 69, **55**, 41 |
| 47 | 15.5 | heptadecane | 240, 99, 85, 71, **57**, 43 |
| 48 | 15.6 | pentadecanal | 208, 180, 110, 96, 82, 69, **57**, 41 |
| 51 | 15.9 | 1,17-octadecadiene | 123, 109, 96, 82, 69, **55**, 41 |
| 52 | 16.0 | 1-octadecene | 252, 125, 111, 97, 83, 69, **55**, 41 |
| 53 | 16.04 | octadecane | 254, 99, 85, 71, **57**, 43 |
| 55 | 16.1 | hexadecanal | 222, 123, 109, 96, 82, 69, **57**, 43 |
| 58 | 16.5 | 1,18-nonadecadiene | 137, 123, 109, 96, 82, 69, **55**, 41 |
| 59 | 16.54 | 1-nonadecene | 139, 125, 111, 97, 83, 69, **55**, 43 |
| 60 | 16.6 | nonadecane | 268, 127, 113, 99, 85, 71, **57**, 43 |
| 62 | 16.7 | heptadecanal | 236, 208, 137, 123, 110, 96, **82**, 68, 57, 41 |
| 64 | 17.0 | 1,19-eicosadiene | 137, 123, 109, 96, 82, 69, **55**, 41 |
| 65 | 17.03 | 1-eicosene | 139, 125, 111, **97**, 83, 69, 55, 43 |
| 66 | 17.06 | eicosane | 127, 113, 99, 85, 71, **57**, 43 |
| 68 | 17.2 | octadecanal | 250, 137, 124, 109, 96, **82**, 68, 57, 43 |
| 70 | 17.4 | 1,20-heneicosadiene | 137, 123, 109, 96, 82, 69, **55**, 41 |
| 71 | 17.5 | 1-heneicosene | 139, 125, 111, **97**, 83, 69, 55, 43 |
| 72 | 17.53 | heneicosane | 113, 99, 85, 71, **57**, 43 |
| 74 | 17.7 | nonadecanal | 264, 202, 137, 124, 110, 96, **82**, 68, 57, 43 |
| 76 | 17.94 | 1,21-docosadiene | 149, 137, 123, 109, 96, 82, 69, **55**, 41 |
| 77 | 17.96 | 1-docosene | 139, 125, 111, **97**, 83, 69, 55, 43 |
| 78 | 17.98 | docosane | 310, 97, 85, 71, **57**, 43 |
| 80 | 18.1 | eicosanal | 278, 250, 124, 109, 96, **82**, 68, 57, 43 |
| 82 | 18.3 | 1,22-tricosadiene | 137, 123, 109, 96, 82, 69, **55**, 41 |
| 83 | 18.40 | 1-tricosene | 322, 125, 111, **97**, 83, 69, 55, 43 |
| 84 | 18.41 | tricosane | 324, 111, 97, 85, 71, **57**, 43 |
| 86 | 18.5 | heneicosanal | 292, 138, 123, 110, 96, **82**, 68, 57, 43 |
| 87 | 18.8 | 1,23-tetracosadiene | 334, 137, 123, 109, 96, 82, 69, **55**, 41 |
| 88 | 18.81 | 1-tetracosene | 336, 139, 125, 111, **97**, 83, 69, 55, 43 |
| 90 | 19.0 | docosanal | 306, 251, 138, 123, 110, 96, **82**, 68, 57, 43 |
| 91 | 19.2 | 1-pentacosene | 350, 139, 125, 111, **97**, 83, 69, 57, 43 |
| 92 | 19.4 | tricosanal | 320, 138, 124, 110, 96, **82**, 68, 57, 43 |
| 93 | 19.6 | 1,25-hexacosadiene | 362, 139, 125, 111, 97, 83, 69, **57**, 43 |
| 102 | 19.8 | unknown | **297**, 149, 105, 77 |

**Table S.2 –** Pyrolysis products identified by Py-GC/MS for the artificially aged HDPE (4 weeks)

|  | t_r_ | Peak identification | Main ions (m/z) |
| --- | --- | --- | --- |
| 1 | 2.7 | 1-hexene | 84, 69, 56, **41** |
| 2 | 4.0 | 1-heptene | 98, 83, 70, **56**, 41 |
| 3 | 4.1 | heptane | 100, 71, 57, **43** |
| 4 | 6.7 | 1-octene | 112, 97, 83, 70, **55**, 41 |
| 5 | 6.9 | octane | 114, 85, 71, 57, **43** |
| 6 | 8.7 | 1,8-nonadiene | 109, 96, 81, **67**, 55, 41 |
| 7 | 8.9 | 1-nonene | 126, 97, 83, 69, **56**, 41 |
| 8 | 9.0 | nonane | 128, 99, 85, 71, 57, **43** |
| 9 | 10.1 | 1,9-decadiene | 110, 95, 81, 67, **55**, 41 |
| 10 | 10.2 | 1-decene | 140, 111, 97, 83, 70, 55, **41** |
| 11 | 10.34 | decane | 142, 113, 99, 85, 71, **57**, 43 |
| 12 | 11.2 | 1,10-undecadiene | 124, 109, 95, 81, 67, 55, **41** |
| 13 | 11.3 | 1-undecene | 154, 126, 111, 97, 83, 70, **55**, 41 |
| 14 | 11.36 | undecane | 156, 98, 85, 71, **57**, 43 |
| 15 | 11.4 | nonanal | 124, 114, 98, 82, 70, **57**, 41 |
| 16 | 12.1 | 1,11-dodecadiene | 138, 124, 109, 95, 81, 67, **55**, 41 |
| 17 | 12.15 | 1-dodecene | 168, 140, 125, 111, 97, 83, 69, 55, **41** |
| 18 | 12.2 | dodecane | 170, 128, 85, 71, **57**, 43 |
| 19 | 12.3 | decanal | 138, 128, 112, 95, 82, 70, 57, **41** |
| 20 | 12.7 | nonanoic acid | 158, 129, 115, 98, **85**, 73, 60, 41 |
| 21 | 12.8 | 1,12-tridecadiene | 123, 109, 95, 81, 67, **55**, 41 |
| 22 | 12.9 | 1-tridecene | 125, 111, 97, 83, 69, 55, **41** |
| 23 | 13.0 | tridecane | 184, 99, 85, 71, **57**, 43 |
| 24 | 13.04 | undecanal | 142, 126, 109, 96, 82, 68, 57, **41** |
| 25 | 13.4 | decanoic acid | 172, 129, **73**, 60, 41 |
| 26 | 13.48 | undecanol | 126, 111, 97, **85**, 69, 55, 41 |
| 27 | 13.5 | 1,13-tetradecadiene | 123, 109, 95, 81, 67, **55**, 41 |
| 28 | 13.6 | 1-tetradecene | 125, 111, 97, 83, 69, **55**, 41 |
| 29 | 13.66 | tetradecane | 198, 99, 85, 71, **57**, 43 |
| 30 | 13.7 | dodecanal | 166, 140, 123, 110, 96, 82, 68, **57**, 41 |
| 31 | 14.06 | undecanoic acid | 186, 143, 129, 115, 85, **73**, 60, 41 |
| 32 | 14.1 | dodecanol | 141, 127, 111, 97, 83, 69, **55**, 43 |
| 33 | 14.2 | 1,14-pentadecadiene | 123, 109, 96, 81, 67, **55**, 41 |
| 34 | 14.26 | 1-pentadecene | 210, 125, 111, 97, 83, 69, **55**, 41 |
| 35 | 14.3 | pentadecane | 141, 127, 113, 99, 85, 71, **57**, 43 |
| 36 | 14.4 | tridecanal | 180, 154, 124, 110, 96, 82, 69, **57**, 41 |
| 37 | 14.6 | dodecanoic acid | 200, 157, 129, 115, 101, 85, **73**, 60, 43 |
| 38 | 14.77 | tridecanol | 182, 155, 141, 125, 111, 97, 83, 69, 55, **43** |
| 39 | 14.8 | 1,15-hexadecadiene | 123, 109, 96, 82, 67, **55**, 41 |
| 40 | 14.88 | 1-hexadecene | 224, 125, 111, 97, 83, 69, **55**, 41 |
| 41 | 14.9 | hexadecane | 226, 99, 85, 71, **57**, 43 |
| 42 | 15.0 | tetradecanal | 194, 168, 138, 124, 109, 96, 82, 69, **57**, 41 |
| 43 | 15.2 | tridecanoic acid | 214, 185, 171, 157, 143, 129, 115, 87, **73**, 60 |
| 44 | 15.36 | tetradecanol | 169, 125, 111, 97, 83, 69, **55**, 43 |
| 45 | 15.4 | 1,16-heptadecadiene | 137, 123, 109, 96, 82, 69, **55**, 41 |
| 46 | 15.45 | 1-heptadecene | 238, 139, 125, 111, 97, 83, 69, **55**, 41 |
| 47 | 15.5 | heptadecane | 240, 99, 85, 71, **57**, 43 |
| 48 | 15.6 | pentadecanal | 208, 182, 109, 96, 82, 69, **57**, 41 |
| 49 | 15.8 | tetradecanoic acid | 228, 185, 129, **73** |
| 50 | 15.93 | pentadecanol | 183, 125, 111, 97, 83, 69, **57**, 41 |
| 51 | 15.96 | 1,17-octadecadiene | 123, 109, 96, 82, 69, **55**, 41 |
| 52 | 16.0 | 1-octadecene | 252, 125, 111, 97, 83, 69, **55**, 41 |
| 53 | 16.04 | octadecane | 254, 99, 85, 71, **57**, 43 |
| 54 | 16.06 | 2-hexadecanone | 240, 111, 97, 85, 71, **58**, 43 |
| 55 | 16.1 | hexadecanal | 222, 123, 109, 96, 82, 69, **57**, 41 |
| 56 | 16.3 | pentadecanoic acid | 242, 199, 129, **73**, 60 |
| 57 | 16.46 | hexadecanol | 197, 125, 111, 97, 83, 69, **55**, 43 |
| 58 | 16.5 | 1,18-nonadecadiene | 137, 123, 109, 96, 82, 69, **55**, 41 |
| 59 | 16.53 | 1-nonadecene | 139, 125, 111, 97, 83, 69, **55**, 43 |
| 60 | 16.55 | nonadecane | 268, 127, 113, 99, 85, 71, **57**, 43 |
| 61 | 16.58 | 2-heptadecanone | 254, 85, 71, **58**, 43 |
| 62 | 16.7 | heptadecanal | 236, 208, 137, 123, 109, 96, **82**, 69, 57, 43 |
| 63 | 16.8 | hexadecanoic acid | 256, 213, 129, **73**, 60 |
| 64 | 16.99 | 1,19-eicosadiene | 137, 123, 109, 96, 82, 69, **55**, 41 |
| 65 | 17.0 | 1-eicosene | 139, 125, 111, **97**, 83, 69, 55, 43 |
| 66 | 17.04 | eicosane | 127, 113, 99, 85, 71, **57**, 43 |
| 67 | 17.09 | 2-octadecanone | 268, 109, 96, 85, **71**, 58, 43 |
| 68 | 17.2 | octadecanal | 250, 137, 123, 109, 96, 82, 69, **57**, 43 |
| 69 | 17.3 | heptadecanoic acid | 270, 227, 185, 171, 129, **73**, 60 |
| 70 | 17.4 | 1,20-heneicosadiene | 137, 123, 109, 96, 82, 69, **55**, 41 |
| 71 | 17.5 | 1-heneicosene | 139, 125, 111, **97**, 83, 69, 55, 43 |
| 72 | 17.52 | heneicosane | 111, 97, 85, 71, **57**, 43 |
| 73 | 17.56 | 2-nonadecanone | 282, 127, 111, 97, 85, 71, **58**, 43 |
| 74 | 17.7 | nonadecanal | 264, 236, 137, 123, 109, 96, **82**, 69, 57, 43 |
| 75 | 17.8 | octadecanoic acid | 284, 241, 185, 129, **73**, 60 |
| 76 | 17.92 | 1,21-docosadiene | 137, 123, 109, 96, 82, 69, **55**, 41 |
| 77 | 17.95 | 1-docosene | 139, 125, 111, **97**, 83, 69, 55, 43 |
| 78 | 17.97 | docosane | 310, 97, 85, 71, **57**, 43 |
| 79 | 18.02 | 2-eicosanone | 297, 127, 96, 85, 71, **58**, 43 |
| 80 | 18.1 | eicosanal | 278, 250, 123, 109, 96, **82**, 69, 57, 43 |
| 81 | 18.2 | nonadecanoic acid | 298, 255, 129, 85, **73** |
| 82 | 18.37 | 1,22-tricosadiene | 137, 123, 111, 97, 83, 69, **55**, 41 |
| 83 | 18.39 | 1-tricosene | 322, 125, 111, **97**, 83, 69, 57, 43 |
| 84 | 18.40 | tricosane | 324, 111, 97, 85, 71, **57**, 43 |
| 85 | 18.46 | 2-heneicosanone | 310, 124, 109, 96, 85, 71, 58, **43** |
| 86 | 18.5 | heneicosanal | 292, 137, 123, 111, 96, **82**, 69, **57**, 43 |
| 87 | 18.78 | 1,23-tetracosadiene | 334, 137, 123, 111, 97, 83, 69, **55**, 43 |
| 88 | 18.8 | 1-tetracosene | 336, 139, 125, 111, **97**, 83, 69, 57, 43 |
| 89 | 18.9 | 2-docosanone | 324, 309, 109, 96, 82, 71, **59**, 43 |
| 90 | 19.0 | docosanal | 306, 250, 137, 125, 111, 97, 82, 71, **57**, 43 |
| 91 | 19.2 | 1-pentacosene | 350, 139, 125, 111, 97, 83, 69, **57**, 43 |
| 92 | 19.4 | tricosanal | 320, 137, 123, 111, 97, 82, 69, **57**, 43 |
| 93 | 19.6 | 1,25-hexacosadiene | 362, 139, 125, 111, 97, 83, 69, **55**, 43 |
| 94 | 19.7 | tetracosanal | 334, 138, 123, 109, 96, **82**, 68, 57, 43 |
| 95 | 20.0 | heptacosane | 380, 125, 111, 97, 85, 71, **57**, 43 |
| 96 | 20.2 | pentacosanal | 348, 123, 111, 97, 83, 69, **57**, 43 |
| 97 | 20.4 | octacosane | 125, 111, 97, 85, 71, **57**, 43 |
| 98 | 20.6 | hexacosanal | 362, 123, 111, 96, 82, 69, **57**, 43 |
| 99 | 20.9 | nonacosane | 139, 125, 111, 97, 83, 71, **57**, 43 |
| 100 | 21.1 | heptacosanal | 376, 123, 111, 96, 82, 71, **57**, 43 |
| 101 | 21.4 | triacontane | 139, 125, 111, 97, 83, 71, **57**, 43 |

**Table S.3 –** Pyrolysis products identified by Py-GC/MS for the unaged LDPE

| # | t_r_ | Peak identification | Main ions (m/z) |
| --- | --- | --- | --- |
| 1 | 2.7 | 1-hexene | 84, 69, **56**, 41 |
| 2 | 3.9 | 1-heptene | 98, 83, 70, **56**, 41 |
| 3 | 4.0 | heptane | 100, 71, 57, **43** |
| 4 | 6.7 | 1-octene | 112, 97, 83, 70, **55**, 41 |
| 5 | 6.9 | octane | 114, 85, 71, 57, **43** |
| 6 | 8.7 | 1,8-nonadiene | 109, 96, 81, **67**, 55, 41 |
| 7 | 8.8 | 1-nonene | 126, 97, 83, 69, **56**, 41 |
| 8 | 9.0 | nonane | 128, 99, 85, 71, 57, **43** |
| 9 | 10.1 | 1,9-decadiene | 110, 95, 81, 67, **55**, 41 |
| 10 | 10.2 | 1-decene | 140, 111, 97, 83, 70, 55, **41** |
| 11 | 10.3 | decane | 142, 117, 99, 85, 71, **57**, 43 |
| 12 | 11.2 | 1,10-undecadiene | 124, 109, 95, 81, 67, 55, **41** |
| 13 | 11.3 | 1-undecene | 154, 126, 111, 97, 83, 70, 55, **43** |
| 14 | 11.4 | undecane | 156, 98, 85, 71, **57**, 43 |
| 15 | 12.1 | 1,11-dodecadiene | 138, 124, 109, 95, 81, 67, **55**, 41 |
| 16 | 12.13 | 1-dodecene | 168, 140, 125, 111, 97, 83, 69, 55, **41** |
| 17 | 12.2 | dodecane | 170, 128, 85, 71, **57**, 43 |
| 18 | 12.3 | decanal | 138, 128, 112, 95, 82, 68, 55, **41** |
| 20 | 12.8 | 1,12-tridecadiene | 123, 109, 95, 81, 67, **55**, 41 |
| 21 | 12.9 | 1-tridecene | 125, 111, 97, 83, 69, 55, **41** |
| 22 | 13.0 | tridecane | 184, 99, 85, 71, **57**, 43 |
| 23 | 13.05 | undecanal | 152, 126, 109, 96, 82, 68, 55, **41** |
| 26 | 13.5 | 1,13-tetradecadiene | 123, 109, 96, 81, 67, **55**, 41 |
| 27 | 13.6 | 1-tetradecene | 125, 111, 97, 83, 69, **55**, 41 |
| 28 | 13.64 | tetradecane | 198, 99, 85, 71, **57**, 43 |
| 29 | 13.72 | dodecanal | 166, 140, 123, 110, 96, 82, 68, **57**, 41 |
| 32 | 14.2 | 1,14-pentadecadiene | 123, 109, 96, 81, 67, **55**, 41 |
| 33 | 14.25 | 1-pentadecene | 210, 125, 111, 97, 83, 69, **55**, 41 |
| 34 | 14.3 | pentadecane | 212, 113, 99, 85, 71, **57**, 43 |
| 35 | 14.4 | tridecanal | 180, 154, 124, 110, 96, 82, 68, **57**, 43 |
| 38 | 14.8 | 1,15-hexadecadiene | 123, 109, 96, 82, 67, **55**, 41 |
| 39 | 14.86 | 1-hexadecene | 224, 125, 111, 97, 83, 69, **55**, 41 |
| 40 | 14.9 | hexadecane | 226, 99, 85, 71, **57**, 43 |
| 41 | 15.0 | tetradecanal | 194, 168, 138, 110, 96, 82, 69, **57**, 41 |
| 44 | 15.4 | 1,16-heptadecadiene | 137, 123, 109, 96, 82, 69, **55**, 41 |
| 45 | 15.44 | 1-heptadecene | 238, 139, 125, 111, 97, 83, 69, **55**, 41 |
| 46 | 15.5 | heptadecane | 240, 99, 85, 71, **57**, 43 |
| 47 | 15.6 | pentadecanal | 208, 180, 111, 96, 82, 69, 57, **41** |
| 50 | 15.9 | 1,17-octadecadiene | 123, 109, 96, 82, 69, **55**, 41 |
| 51 | 16.0 | 1-octadecene | 252, 125, 111, **97**, 83, 69, 55, 41 |
| 52 | 16.02 | octadecane | 254, 99, 85, 71, **57**, 43 |
| 54 | 16.1 | hexadecanal | 222, 124, 111, 96, 82, 69, **57**, 43 |
| 57 | 16.5 | 1,18-nonadecadiene | 137, 123, 109, 96, 82, 69, **55**, 41 |
| 58 | 16.51 | 1-nonadecene | 139, 125, 111, 97, **83**, 69, 55, 43 |
| 59 | 16.54 | nonadecane | 268, 127, 113, 99, 85, 71, **57**, 43 |
| 61 | 16.7 | heptadecanal | 236, 208, 137, 124, 109, 96, **82**, 68, 57, 41 |
| 63 | 16.97 | 1,19-eicosadiene | 137, 123, 109, 96, 82, 69, **55**, 41 |
| 64 | 17.01 | 1-eicosene | 139, 125, 111, **97**, 83, 69, 55, 43 |
| 65 | 17.04 | eicosane | 127, 113, 99, 85, 71, **57**, 43 |
| 67 | 17.1 | octadecanal | 250, 137, 124, 109, 96, **82**, 68, 57, 43 |
| 69 | 17.4 | 1,20-heneicosadiene | 137, 123, 109, 96, 82, 69, **55**, 41 |
| 70 | 17.48 | 1-heneicosene | 139, 125, 111, **97**, 83, 69, 55, 43 |
| 71 | 17.50 | heneicosane | 113, 97, 85, 71, **57**, 43 |
| 73 | 17.6 | nonadecanal | 264, 202, 137, 124, 110, 97, **82**, 69, 57, 43 |
| 75 | 17.91 | 1,21-docosadiene | 151, 137, 123, 109, 96, 82, 67, **55**, 41 |
| 76 | 17.94 | 1-docosene | 139, 125, 111, **97**, 83, 69, 55, 43 |
| 77 | 17.96 | docosane | 310, 99, 85, 71, **57**, 43 |
| 79 | 18.1 | eicosanal | 278, 250, 123, 111, 96, 82, 69, **57**, 43 |
| 81 | 18.3 | 1,22-tricosadiene | 137, 123, 109, 96, 82, 69, **55**, 41 |
| 82 | 18.37 | 1-tricosene | 322, 125, 111, **97**, 83, 69, 55, 43 |
| 83 | 18.39 | tricosane | 324, 113, 97, 85, 71, **57**, 43 |
| 85 | 18.5 | heneicosanal | 292, 138, 123, 111, 96, 82, 68, **57**, 43 |
| 86 | 18.77 | 1,23-tetracosadiene | 334, 137, 123, 109, 96, 82, 69, **55**, 41 |
| 87 | 18.8 | 1-tetracosene | 336, 139, 125, 111, **97**, 83, 69, 57, 43 |
| 89 | 19.0 | docosanal | 306, 251, 138, 124, 111, 96, 82, 71, **57**, 43 |
| 90 | 19.2 | 1-pentacosene | 350, 139, 125, 111, **97**, 83, 69, 57, 43 |
| 91 | 19.35 | tricosanal | 320, 139, 125, 111, 97, 83, 71, **57**, 43 |
| 92 | 19.6 | 1-hexacosene | 364, 139, 125, 111, **97**, 83, 69, 57, 43 |
| 103 | 19.8 | unknown | **297**, 149, 105, 77 |

**Table S.4 –** Pyrolysis products identified by Py-GC/MS for the artificially aged LDPE (4 weeks)

| # | t_r_ | Peak identification | Main ions (m/z) |
| --- | --- | --- | --- |
| 1 | 2.7 | 1-hexene | 84, 69, 56, 41 |
| 2 | 3.9 | 1-heptene | 98, 83, 70, **56**, 41 |
| 3 | 4.0 | heptane | 100, 71, 57, **43** |
| 4 | 6.7 | 1-octene | 112, 97, 83, 70, **55**, 41 |
| 5 | 6.9 | octane | 114, 85, 71, 57, **43** |
| 6 | 8.7 | 1,8-nonadiene | 109, 96, 81, **67**, 55, 41 |
| 7 | 8.9 | 1-nonene | 126, 97, 83, 69, **56**, 41 |
| 8 | 9.0 | nonane | 128, 99, 85, 71, 57, **43** |
| 9 | 10.1 | 1,9-decadiene | 110, 95, 81, 67, **55**, 41 |
| 10 | 10.2 | 1-decene | 140, 111, 97, 83, 70, 55, **41** |
| 11 | 10.35 | decane | 142, 113, 99, 85, 71, **57**, 43 |
| 12 | 11.2 | 1,10-undecadiene | 124, 109, 95, 81, 67, 55, **41** |
| 13 | 11.3 | 1-undecene | 154, 126, 111, 97, 83, 70, 55, **41** |
| 14 | 11.36 | undecane | 156, 98, 85, 71, **57**, 43 |
| 15 | 12.1 | 1,11-dodecadiene | 138, 124, 109, 95, 81, 67, **55**, 41 |
| 16 | 12.15 | 1-dodecene | 168, 140, 125, 111, 97, 83, 69, 55, **41** |
| 17 | 12.2 | dodecane | 170, 127, 85, 71, **57**, 43 |
| 18 | 12.3 | decanal | 138, 128, 112, 95, 82, 70, 57, **41** |
| 19 | 12.7 | nonanoic acid | 158, 129, 115, 98, 85, 73, **57**, 41 |
| 20 | 12.8 | 1,12-tridecadiene | 123, 109, 95, 81, 67, **55**, 41 |
| 21 | 12.9 | 1-tridecene | 125, 111, 97, 83, 69, **55**, 41 |
| 22 | 13.0 | tridecane | 184, 99, 85, 71, **57**, 43 |
| 23 | 13.03 | undecanal | 142, 126, 109, 96, 82, 68, 57, **41** |
| 24 | 13.4 | decanoic acid | 172, 129, **73**, 55, 41 |
| 25 | 13.48 | undecanol | 126, 111, 97, **85**, 69, 55, 43 |
| 26 | 13.5 | 1,13-tetradecadiene | 123, 109, 96, 81, 67, **55**, 41 |
| 27 | 13.6 | 1-tetradecene | 125, 111, 97, 83, 69, **55**, 41 |
| 28 | 13.66 | tetradecane | 198, 99, 85, 71, **57**, 43 |
| 29 | 13.7 | dodecanal | 166, 140, 123, 110, 96, 82, 68, **57**, 41 |
| 30 | 14.05 | undecanoic acid | 186, 143, 129, 115, 83, **73**, 60, 41 |
| 31 | 14.1 | dodecanol | 141, 127, 111, 97, 83, 69, **57**, 41 |
| 32 | 14.2 | 1,14-pentadecadiene | 123, 109, 96, 82, 67, **55**, 41 |
| 33 | 14.26 | 1-pentadecene | 210, 125, 111, 97, 83, 69, **55**, 41 |
| 34 | 14.3 | pentadecane | 141, 127, 113, 99, 85, 71, **57**, 43 |
| 35 | 14.4 | tridecanal | 180, 154, 124, 110, 96, 82, 68, **57**, 41 |
| 36 | 14.6 | dodecanoic acid | 200, 157, 129, 115, 98, 85, **73**, 60, 43 |
| 37 | 14.77 | tridecanol | 182, 155, 141, 125, 111, 97, 83, 69, 55, **43** |
| 38 | 14.8 | 1,15-hexadecadiene | 123, 109, 96, 82, 69, **55**, 41 |
| 39 | 14.87 | 1-hexadecene | 224, 125, 111, 97, 83, 69, **55**, 41 |
| 40 | 14.9 | hexadecane | 226, 99, 85, 71, **57**, 43 |
| 41 | 15.0 | tetradecanal | 194, 168, 138, 124, 109, 96, 82, 69, **57**, 41 |
| 42 | 15.2 | tridecanoic acid | 214, 185, 171, 157, 143, 129, 115, 85, **73**, 60 |
| 43 | 15.37 | tetradecanol | 169, 125, 111, 97, 83, 69, **55**, 41 |
| 44 | 15.4 | 1,16-heptadecadiene | 137, 123, 109, 96, 82, 69, **55**, 41 |
| 45 | 15.45 | 1-heptadecene | 238, 139, 125, 111, 97, **83**, 69, 55, 41 |
| 46 | 15.5 | heptadecane | 240, 99, 85, 71, **57**, 43 |
| 47 | 15.6 | pentadecanal | 208, 182, 109, 96, **82**, 69, 57, 43 |
| 48 | 15.8 | tetradecanoic acid | 228, 185, 129, **73** |
| 49 | 15.93 | pentadecanol | 183, 125, 111, 97, 83, 70, **57**, 41 |
| 50 | 15.96 | 1,17-octadecadiene | 123, 109, 96, 82, 69, **55**, 41 |
| 51 | 16.0 | 1-octadecene | 252, 125, 111, 97, **83**, 69, 55, 43 |
| 52 | 16.03 | octadecane | 254, 99, 85, 71, **57**, 43 |
| 53 | 16.05 | 2-hexadecanone | 240, 111, 97, 85, 71, 58, **43** |
| 54 | 16.1 | hexadecanal | 222, 123, 109, 96, **82**, 68, 57, 43 |
| 55 | 16.3 | pentadecanoic acid | 242, 199, 129, **73**, 57 |
| 56 | 16.46 | hexadecanol | 197, 125, 111, 97, 83, 69, **55**, 41 |
| 57 | 16.5 | 1,18-nonadecadiene | 137, 123, 109, 96, 82, 69, **55**, 41 |
| 58 | 16.53 | 1-nonadecene | 139, 125, 111, **97**, 83, 69, 55, 43 |
| 59 | 16.55 | nonadecane | 268, 127, 113, 99, 85, 71, **57**, 43 |
| 60 | 16.58 | 2-heptadecanone | 254, 85, 71, **58**, 43 |
| 61 | 16.7 | heptadecanal | 236, 208, 137, 123, 109, 96, **82**, 68, 57, 43 |
| 62 | 16.8 | hexadecanoic acid | 256, 213, 129, **73**, 55, **43** |
| 63 | 16.99 | 1,19-eicosadiene | 137, 123, 109, 96, 82, 69, **55**, 41 |
| 64 | 17.0 | 1-eicosene | 139, 125, 111, **97**, 83, 69, 55, 43 |
| 65 | 17.04 | eicosane | 127, 113, 99, 85, 71, **57**, 43 |
| 66 | 17.09 | 2-octadecanone | 268, 114, 96, 85, 71, **58**, 43 |
| 67 | 17.2 | octadecanal | 250, 137, 124, 109, 96, **82**, 68, 57, 43 |
| 68 | 17.3 | heptadecanoic acid | 270, 227, 185, 171, 129, **70**, **55** |
| 69 | 17.47 | 1,20-heneicosadiene | 137, 123, 109, 96, 82, 69, **55**, 41 |
| 70 | 17.5 | 1-heneicosene | 139, 125, 111, **97**, 83, 69, 55, 43 |
| 71 | 17.52 | heneicosane | 113, 99, 85, 71, **57**, 43 |
| 72 | 17.56 | 2-nonadecanone | 282, 127, 113, 95, 85, 71, **58**, 43 |
| 73 | 17.65 | nonadecanal | 264, 236, 137, 123, 109, 96, **82**, 69, 57, 43 |
| 74 | 17.8 | octadecanoic acid | 284, 241, 185, 129, 97, **57** |
| 75 | 17.92 | 1,21-docosadiene | 137, 123, 109, 96, 82, 69, **55**, 41 |
| 76 | 17.95 | 1-docosene | 139, 125, 111, **97**, 83, 69, 57, 43 |
| 77 | 17.97 | docosane | 310, 99, 85, 71, **57**, 43 |
| 78 | 18.02 | 2-eicosanone | 296, 127, 96, 85, 71, **58**, 43 |
| 79 | 18.1 | eicosanal | 278, 250, 123, 109, 96, **82**, 69, 57, 43 |
| 80 | 18.2 | nonadecanoic acid | 298, 255, 129, 83, 69, **57**, 43 |
| 81 | 18.36 | 1,22-tricosadiene | 137, 123, 109, 96, 82, 69, **55**, 41 |
| 82 | 18.39 | 1-tricosene | 322, 125, 111, **97**, 83, 69, 57, 43 |
| 83 | 18.40 | tricosane | 324, 113, 97, 85, 71, **57**, 43 |
| 84 | 18.46 | 2-heneicosanone | 310, 127, 109, 96, 82, 71, **59**, 43 |
| 85 | 18.5 | heneicosanal | 292, 138, 123, 111, 96, **82**, 69, 57, 43 |
| 86 | 18.78 | 1,23-tetracosadiene | 334, 137, 123, 109, 96, 82, 69, **55**, 41 |
| 87 | 18.8 | 1-tetracosene | 336, 139, 125, 111, **97**, 83, 69, 57, 43 |
| 88 | 18.9 | 2-docosanone | 324, 309, 109, 96, 82, 71, **58**, 43 |
| 89 | 19.0 | docosanal | 306, 250, 138, 123, 109, 96, **82**, 69, 57, 43 |
| 90 | 19.2 | 1-pentacosene | 350, 139, 125, 111, **97**, 83, 69, 57, 43 |
| 91 | 19.4 | tricosanal | 320, 137, 124, 109, 96, **82**, 68, 57, 43 |
| 92 | 19.6 | 1-hexacosene | 364, 139, 125, 111, 97, 83, 69, **57**, 43 |
| 93 | 19.8 | tetracosanal | 334, 138, 123, 109, 96, **82**, 69, 57, 43 |
| 94 | 20.0 | heptacosane | 380, 125, 111, 97, 85, 71, **57**, 43 |
| 95 | 20.2 | pentacosanal | 348, 125, 111, 97, 83, 71, **57**, 43 |
| 96 | 20.4 | octacosane | 125, 111, 97, 83, 71, **57**, 43 |
| 97 | 20.6 | hexacosanal | 362, 123, 109, 96, 82, 69, **57**, 43 |
| 98 | 20.9 | nonacosane | 139, 125, 111, 97, 83, 69, **57**, 43 |
| 99 | 21.1 | heptacosanal | 376, 123, 111, 96, 82, 71, **57**, 43 |
| 100 | 21.4 | triacontane | 139, 125, 111, 97, 83, 71, **57**, 43 |
| 101 | 22.0 | hentriacontane | 139, 125, 111, 97, 83, 71, **57**, 43 |
| 102 | 22.7 | dotriacontane | 125, 111, 97, 85, 71, **57**, 43 |

**Table S.5 –** Pyrolysis products identified by Py-GC/MS for the unaged PP

| # | t_r_ | Peak identification | Main ions (m/z) |
| --- | --- | --- | --- |
| 1 | 1.9 | propene | 42, **41**, 39 |
| 2 | 2.2 | pentane | 72, 57, **43** |
| 3 | 2.7 | 2,methyl-1-pentene | 84, 69, **56**, 41 |
| 4 | 5.6 | 4-methyl-2-heptene | 112, **69**, 55, 41 |
| 5 | 6.2 | 2-methyl-1,5-hexadiene | **95**, 81, 67, 55, 39 |
| 6 | 8 | 2,4-dimethyl-1-heptene | 126, 83, 70, 55, **43** |
| 7 | 8.2 | 1,3,5-trimethylcyclohexane (isomer) | 126, **111**, 69, 55, 41 |
| 8 | 8.5 | 2,4-dimethyl-1,6-heptadiene | 124, **109**, 81, 67, 55, 41 |
| 9 | 10.3 | 4,6-dimethyl-2-nonene | 154, 111, 85, **69**, 55, 41 |
| 10 | 11.1 | 2,4,6-trimethyl-1-nonene (meso form) | 168, 125, 111, 83, **69**, 57, 43 |
| 11 | 11.2 | 2,4,6-trimethyl-1-nonene (racemic form) | 168, 125, 111, 83, **69**, 57, 43 |
| 12 | 11.6 | 2,4,6,8-tetramethyl-1-nonene (racemic form) | 182, 125, 111, 83, **69**, 57, 43 |
| 13 | 11.8 | 2,4,6,8-tetramethyl-1,8-nonadiene (racemic form) | 180, 165, 123, 109, 96, 83, **69**, 55, 41 |
| 14 | 13.0 | 2,4,6,8-tetramethyl-1-undecene (isotactic) | 210, 154, 111, 83, **69**, 55, 43 |
| 15 | 13.1 | 2,4,6,8-tetramethyl-1-undecene (heterotactic) | 210, 154, 111, 85, **69**, 55, 43 |
| 16 | 13.2 | 2,4,6,8-tetramethyl-1-undecene (syndiotactic) | 210, 154, 111, 85, **69**, 55, 44 |
| 17 | 13.3 | 2,4,6,8,10-pentamethyl-1-undecene (syndiotactic) | 224, 168, 125, 111, 97, 83, **69**, 57, 43 |
| 18 | 13.6 | 2,4,6,8,10-pentamethyl-1,10-undecadiene | 222, 154, 137, 123, 109, 95, 83, **69**, 55, 41 |
| 19 | 13.9 | unknown | 153, 125, 111, 97, 85, **69**, 57, 43 |
| 20 | 14.3 | 2,4,6,8,10-pentamethyl-1,14-tridecadiene | 250, 123, 109, 95, 83, **69**, 55, 41 |
| 21 | 14.5 | 2,4,6,8,10-pentamethyl-1-tridecene (isotactic) | 252, 153, 139, 125, 111, 97, 83, **69**, 57, 43 |
| 22 | 14.6 | unknown | 196, 153, 125, 111, 97, 83, **69**, 57, 43 |
| 23 | 14.7 | unknown | 153, 125, 111, 97, 83, **69**, 57, 43 |
| 24 | 14.9 | 2,4,6,8,10,12-hexamethyl-1,12-tridecadiene (syndiotactic) | 264, 165, 151, 137, 123, 109, 95, 83, **69**, 55, 41 |
| 25 | 15.6 | 2,4,6,8,10,12-hexamethyl-1,14-pentadecadiene | 292, 151, 137, 123, 109, 97, 83, **69**, 55, 41 |
| 26 | 15.7 | 2,4,6,8,10,12-hexamethyl-1-pentadecene | 294, 153, 139, 125, 111, 97, 83, **69**, 57, 43 |
| 27 | 15.9 | unknown | 294, 153, 139, 125, 111, 97, 83, **69**, 57, 43 |
| 28 | 16.1 | 2,4,6,8,10,12,14-heptamethyl-1,14-pentadecadiene | 306, 165, 151, 137, 123, 109, 97, 83, **69**, 55, 41 |
| 29 | 16.8 | 2,4,6,8,10,12,14-heptamethyl-1,16-heptadecadiene | 334, 151, 137, 123, 109, 97, 83, **69**, 55, 41 |
| 30 | 16.9 | 2,4,6,8,10,12,14-heptamethyl-1-heptadecene | 336, 153, 139, 125, 111, 97, 83, **69**, 57, 43 |
| 31 | 17.0 | unknown | 153, 125, 111, 97, 83, **69**, 57, 43 |
| 32 | 17.1 | unknown | 153, 125, 111, 97, 83, **69**, 57, 43 |
| 33 | 17.2 | 2,4,6,8,10,12,14,16-octamethyl-1,16-eptadecadiene | 348, 165, 151, 137, 123, 109, 97, 83, **69**, 55, 41 |
| 34 | 17.8 | 2,4,6,8,10,12,14,16-octamethyl-1,18-nonadecadiene | 376, 151, 137, 125, 109, 97, 83, **69**, 55, 41 |
| 35 | 17.9 | 2,4,6,8,10,12,14,16-octamethyl-1-nonadecene | 378, 153, 139, 125, 111, 97, 83, **69**, 57, 43 |
| 36 | 18.2 | 2,4,6,8,10,12,14,16,18-nonamethyl-1,18-nonadecadiene | 390, 165, 151, 137, 125, 109, 97, 83, **69**, 55, 41 |
| 37 | 18.7 | 2,4,6,8,10,12,14,16,18-nonamethyl-1,20-henicosadiene | 418, 153, 125, 111, 97, 83, **69**, 55, 41 |
| 38 | 18.8 | 2,4,6,8,10,12,14,16,18-nonamethyl-1-henicosene | 420, 153, 139, 125, 111, 97, 83, **69**, 57, 43 |
| 39 | 19.1 | 2,4,6,8,10,12,14,16,18,20-decamethyl-1,20-henicosadiene | 432, 165, 153,137, 125, 109, 97, 83, **69**, 55, 43 |
| 40 | 19.6 | 2,4,6,8,10,12,14,16,18,20-decamethyl-1,22-tricosadiene | 460, 153, 125, 111, 97,83, **69**, 55, 41 |
| 41 | 19.65 | 2,4,6,8,10,12,14,16,18,20-decamethyl-1-tricosene | 153, 139, 125, 111, 97, 83, **69**, 57, 43 |
| 42 | 20.0 | 2,4,6,8,10,12,14,16,18,20,22-undecamethyl-1,22-tricosadiene | 474, 125, 111, 97, 83, **69**, 55, 43 |

**Table S.6 –** Pyrolysis products identified by Py-GC/MS for the artificially aged PP (4 weeks)

| # | t_r_ | Peak identification | Main ions (m/z) |
| --- | --- | --- | --- |
| 1 | 1.9 | propene | 42, **41**, 39 |
| 2 | 2.2 | pentane | 72, 57, **43** |
| 3 | 2.7 | 2,methyl-1-pentene | 84, 69, **56**, 41 |
| 4 | 5.6 | 4-methyl-2-heptene | 112, **69**, 55, 41 |
| 5 | 6.2 | 2-methyl-1,5-hexadiene | **95**, 81, 67, 55, 39 |
| 6 | 8 | 2,4-dimethyl-1-heptene | 126, 83, 70, 55, **43** |
| 7 | 8.2 | 1,3,5-trimethylcyclohexane (isomer) | 126, **111**, 69, 55, 41 |
| 8 | 8.5 | 2,4-dimethyl-1,6-heptadiene | 124, **109**, 81, 67, 55, 41 |
| 9 | 10.3 | 4,6-dimethyl-2-nonene | 154, 111, 85, **69**, 55, 41 |
| 10 | 11.1 | 2,4,6-trimethyl-1-nonene (meso form) | 168, 125, 111, 83, **69**, 57, 43 |
| 11 | 11.2 | 2,4,6-trimethyl-1-nonene (racemic form) | 168, 125, 111, 83, **69**, 57, 43 |
| 12 | 11.6 | 2,4,6,8-tetramethyl-1-nonene (racemic form) | 182, 125, 111, 83, **69**, 57, 43 |
| 13 | 11.8 | 2,4,6,8-tetramethyl-1,8-nonadiene (racemic form) | 180, 165, 123, 109, 96, 83, **69**, 55, 41 |
| 14 | 13.0 | 2,4,6,8-tetramethyl-1-undecene (isotactic) | 210, 154, 111, 83, **69**, 55, 43 |
| 15 | 13.1 | 2,4,6,8-tetramethyl-1-undecene (heterotactic) | 210, 154, 111, 85, **69**, 55, 43 |
| 16 | 13.2 | 2,4,6,8-tetramethyl-1-undecene (syndiotactic) | 210, 154, 111, 85, **69**, 55, 44 |
| 17 | 13.3 | 2,4,6,8,10-pentamethyl-1-undecene (syndiotactic) | 224, 168, 125, 111, 97, 83, **69**, 57, 43 |
| 18 | 13.6 | 2,4,6,8,10-pentamethyl-1,10-undecadiene | 222, 154, 137, 123, 109, 95, 83, **69**, 55, 41 |
| 19 | 13.9 | unknown | 153, 125, 111, 97, 85, **69**, 57, 43 |
| 20 | 14.3 | 2,4,6,8,10-pentamethyl-1,14-tridecadiene | 250, 123, 109, 95, 83, **69**, 55, 41 |
| 21 | 14.5 | 2,4,6,8,10-pentamethyl-1-tridecene (isotactic) | 252, 153, 139, 125, 111, 97, 83, **69**, 57, 43 |
| 22 | 14.6 | unknown | 196, 153, 125, 111, 97, 83, **69**, 57, 43 |
| 23 | 14.7 | unknown | 153, 125, 111, 97, 83, **69**, 57, 43 |
| 24 | 14.9 | 2,4,6,8,10,12-hexamethyl-1,12-tridecadiene (syndiotactic) | 264, 165, 151, 137, 123, 109, 95, 83, **69**, 55, 41 |
| 25 | 15.6 | 2,4,6,8,10,12-hexamethyl-1,14-pentadecadiene | 292, 151, 137, 123, 109, 97, 83, **69**, 55, 41 |
| 26 | 15.7 | 2,4,6,8,10,12-hexamethyl-1-pentadecene | 294, 153, 139, 125, 111, 97, 83, **69**, 57, 43 |
| 27 | 15.9 | unknown | 294, 153, 139, 125, 111, 97, 83, **69**, 57, 43 |
| 28 | 16.1 | 2,4,6,8,10,12,14-heptamethyl-1,14-pentadecadiene | 306, 165, 151, 137, 123, 109, 97, 83, **69**, 55, 41 |
| 29 | 16.8 | 2,4,6,8,10,12,14-heptamethyl-1,16-heptadecadiene | 334, 151, 137, 123, 109, 97, 83, **69**, 55, 41 |
| 30 | 16.9 | 2,4,6,8,10,12,14-heptamethyl-1-heptadecene | 336, 153, 139, 125, 111, 97, 83, **69**, 57, 43 |
| 31 | 17.0 | unknown | 153, 125, 111, 97, 83, **69**, 57, 43 |
| 32 | 17.1 | unknown | 153, 125, 111, 97, 83, **69**, 57, 43 |
| 33 | 17.2 | 2,4,6,8,10,12,14,16-octamethyl-1,16-eptadecadiene | 348, 165, 151, 137, 123, 109, 97, 83, **69**, 55, 41 |
| 34 | 17.8 | 2,4,6,8,10,12,14,16-octamethyl-1,18-nonadecadiene | 376, 151, 137, 125, 109, 97, 83, **69**, 55, 41 |
| 35 | 17.9 | 2,4,6,8,10,12,14,16-octamethyl-1-nonadecene | 378, 153, 139, 125, 111, 97, 83, **69**, 57, 43 |
| 36 | 18.2 | 2,4,6,8,10,12,14,16,18-nonamethyl-1,18-nonadecadiene | 390, 165, 151, 137, 125, 109, 97, 83, **69**, 55, 41 |
| 37 | 18.7 | 2,4,6,8,10,12,14,16,18-nonamethyl-1,20-henicosadiene | 418, 153, 125, 111, 97, 83, **69**, 55, 41 |
| 38 | 18.8 | 2,4,6,8,10,12,14,16,18-nonamethyl-1-henicosene | 420, 153, 139, 125, 111, 97, 83, **69**, 57, 43 |
| 39 | 19.1 | 2,4,6,8,10,12,14,16,18,20-decamethyl-1,20-henicosadiene | 432, 165, 153,137, 125, 109, 97, 83, **69**, 55, 43 |
| 40 | 19.6 | 2,4,6,8,10,12,14,16,18,20-decamethyl-1,22-tricosadiene | 460, 153, 125, 111, 97,83, **69**, 55, 41 |
| 41 | 19.65 | 2,4,6,8,10,12,14,16,18,20-decamethyl-1-tricosene | 153, 139, 125, 111, 97, 83, **69**, 57, 43 |
| 42 | 20.0 | 2,4,6,8,10,12,14,16,18,20,22-undecamethyl-1,22-tricosadiene | 474, 125, 111, 97, 83, **69**, 55, 43 |

**Table S.7 –** Pyrolysis products identified by Py-GC/MS for the unaged PS

| # | t_r_ | Peak identification | Main ions (m/z) |
| --- | --- | --- | --- |
| 1 | 6.0 | toluene | 92, **91**, 65 |
| 2 | 9.0 | styrene | **104**, 78, 63, 51 |
| 3 | 9.7 | allylbenzene | **117**, 103, 91, 65 |
| 4 | 9.9 | benzaldehyde | 106, **105**, 77, 51 |
| 5 | 10.2 | α-methylstyrene | **118**, 103, 91, 78, 63, 51 |
| 6 | 10.9 | 3-butenylbenzene | 132, 104, **91**, 65 |
| 7 | 11.0 | (1-methylenepropyl)-benzene | 132, **117**, 103, 91, 77, 63, 51 |
| 8 | 11.1 | acetophenone | 120, **105**, 91, 77, 51 |
| 9 | 14.6 | 1,2-diphenylethane | 182, **91**, 65 |
| 10 | 14.8 | propane-1,2-diyldibenzene | 196, **105**, 91, 77, 65 |
| 11 | 15.4 | 1,1'-(1,3-propanediyl)bis-benzene | 196, 105, **92**, 77, 65, 51 |
| 12 | 15.7 | Stilbene | 180, **179**, 165, 152, 102, 89, 76 |
| 13 | 15.8 | 3-butene-1,3-diyldibenzene (styrene dimer) | 208, 193, 130, 115, 104, **91**, 77, 65 |
| 14 | 15.9 | 1-pentene-2,4-diyldibenzen | 222, 194, 179, 115, **105**, 91, 77 |
| 15 | 16.3 | (E)-1-butene-1,4-diyldibenzene | 208, **117,** 115, 91, 65 |
| 16 | 16.7 | hexa-1,5-diene-2,5-diyldibenzene | 234, 143, **130**, 115, 104, 91, 77, 65 |
| 17 | 19.2 | 5-hexene-1,3,5-triyltribenzene (styrene trimer) | 312, 207, 194, 117, **91**, 77 |

**Table S.8 –** Pyrolysis products identified by Py-GC/MS for the artificially aged PS (4 weeks)

| # | t_r_ | Peak identification | Main ions (m/z) |
| --- | --- | --- | --- |
| 1 | 6.0 | toluene | 92, **91**, 65 |
| 2 | 8.9 | styrene | **104**, 78, 63, 51 |
| 3 | 9.7 | allylbenzene | **117**, 103, 91, 65 |
| 4 | 9.9 | benzaldehyde | 106, **105**, 77, 51 |
| 5 | 10.2 | α-methylstyrene | **118**, 103, 91, 78, 63, 51 |
| 6 | 10.8 | 3-butenylbenzene | 132, 104, **91**, 65 |
| 7 | 11.0 | (1-methylenepropyl)-benzene | 132, **117**, 103, 91, 77, 63, 51 |
| 8 | 11.1 | acetophenone | 120, **105**, 91, 77, 51 |
| 9 | 14.6 | 1,2-diphenylethane | 182, **91**, 65 |
| 10 | 14.8 | propane-1,2-diyldibenzene | 196, **105**, 91, 77, 65 |
| 11 | 15.4 | 1,1'-(1,3-propanediyl)bis-benzene | 196, 105, **92**, 77, 65, 51 |
| 12 | 15.7 | Stilbene | 180, **179**, 165, 152, 102, 89, 76 |
| 13 | 15.8 | 3-butene-1,3-diyldibenzene (styrene dimer) | 208, 193, 130, 115, 104, **91**, 77, 65 |
| 14 | 15.9 | 1-pentene-2,4-diyldibenzen | 222, 194, 179, 115, **105**, 91, 77 |
| 15 | 16.3 | (E)-1-butene-1,4-diyldibenzene | 208, **117,** 115, 91, 65 |
| 16 | 16.7 | hexa-1,5-diene-2,5-diyldibenzene | 234, 143, **130**, 115, 104, 91, 77, 65 |
| 17 | 19.2 | 5-hexene-1,3,5-triyltribenzene (styrene trimer) | 312, 207, 194, 117, **91**, 77 |

**Table S.9 –** Pyrolysis products identified by Py-GC/MS for the unaged PET

| # | t_r_ | Peak identification | Main ions (m/z) |
| --- | --- | --- | --- |
| 1 | 2.0 | carbon dioxide | **44** |
| 2 | 3.5 | benzene | **78**, 63, 51 |
| 3 | 8.3 | ethylbenzene | 106, **91**, 77, 65, 51 |
| 4 | 8.9 | styrene | **104**, 78, 63, 51 |
| 5 | 9.9 | benzaldehyde | 106, **105**, 77, 51 |
| 6 | 10.1 | phenol | **94**, 79, 66, 55, 39 |
| 7 | 10.9 | benzeneacetaldehyde | 120, **91**, 65, 51 |
| 8 | 11.1 | acetophenone | 120, **105**, 77, 51 |
| 9 | 11.8 | vinyl benzoate | 148, **105**, 77, 51 |
| 10 | 12.4 | benzoic acid | 122, **105**, 77, 51 |
| 11 | 13.7 | biphenyl | **154**, 131, 115, 76 |
| 12 | 14.8 | divinyl terephthalate | **175**, 147, 132, 104, 76 |
| 13 | 15.1 | 4-(vinyloxycarbonyl) benzoic acid | **149**, 12176, 65 |
| 14 | 15.2 | benzophenone | 182, 152, **105**, 77, 51 |
| 15 | 15.7 | stilbene | **180**, 179, 165, 152, 102, 89, 76 |
| 16 | 15.9 | 9H-fluoren-9-one | **180**, 152, 126, 76 |
| 17 | 16.7 | unknown | 198, **181**, 152, 76 |
| 18 | 17.9 | ethan-1,2-diyldibenzoate | 227, **105**, 77, 51 |
| 19 | 18.2 | unknown | **230**, 198, 181, 152, 115 |
| 20 | 19.0 | unknown | 296, 149, **131**, 105, 77, 51 |
| 21 | 19.8 | 2-(benzoyloxy) ethyl vinyl terephthalate | **297**, 149, 105, 77 |
| 22 | 22.0 | ethan-1,2-diyl divinyl diterephthalate | **367**, 325, 296, 175, 162, 104 |

**Table S.10 –** Pyrolysis products identified by Py-GC/MS for the artificially aged PET (4 weeks)

| # | t_r_ | Peak identification | Main ions (m/z) |
| --- | --- | --- | --- |
| 1 | 2.0 | carbon dioxide | **44** |
| 2 | 3.5 | benzene | **78**, 63, 51 |
| 3 | 8.3 | ethylbenzene | 106, **91**, 77, 65, 51 |
| 4 | 8.8 | styrene | **104**, 78, 63, 51 |
| 5 | 9.9 | benzaldehyde | 106, **105**, 77, 51 |
| 6 | 10.1 | phenol | **94**, 79, 66, 55, 39 |
| 7 | 10.8 | benzeneacetaldehyde | 120, **91**, 65, 51 |
| 8 | 11.1 | acetophenone | 120, **105**, 77, 51 |
| 9 | 11.7 | vinyl benzoate | 148, **105**, 77, 51 |
| 10 | 12.4 | benzoic acid | 122, **105**, 77, 51 |
| 11 | 13.7 | biphenyl | **154**, 131, 115, 76 |
| 12 | 14.8 | divinyl terephthalate | **175**, 147, 132, 104, 76 |
| 13 | 15.0 | 4-(vinyloxycarbonyl) benzoic acid | **149**, 12176, 65 |
| 14 | 15.2 | benzophenone | 182, 152, **105**, 77, 51 |
| 15 | 15.7 | stilbene | **180**, 179, 165, 152, 102, 89, 76 |
| 16 | 15.9 | 9H-fluoren-9-one | **180**, 152, 126, 76 |
| 17 | 16.7 | unknown | 198, **181**, 152, 76 |
| 18 | 17.9 | ethan-1,2-diyldibenzoate | 227, **105**, 77, 51 |
| 19 | 18.2 | unknown | **230**, 198, 181, 152, 115 |
| 20 | 19.0 | unknown | 296, 149, **131**, 105, 77, 51 |
| 21 | 19.8 | 2-(benzoyloxy) ethyl vinyl terephthalate | **297**, 149, 105, 77 |
| 22 | 22.0 | ethan-1,2-diyl divinyl diterephthalate | **367**, 325, 296, 175, 162, 104 |

1. Jung MR, et al. (2018). Validation of ATR FT-IR to identify polymers of plastic marine debris, including those ingested by marine organisms. Mar Pollut Bull 127, 704-716.

   Renner G, Schmidt TC, Schram J, Characterization and Quantification of Microplastics by Infrared Spectroscopy; in “Characterization and Analysis of Microplastics”, Rocha-Santos TAP, Duarte AC Eds., Elsevier (2017), Comprehensive Anal Chem 75, Ch. 4 p. 67-118. [↑](#footnote-ref-1)
